# New advances of NG2-expressing cell subset in marrow mesenchymal stem cells as novel therapeutic tools for liver fibrosis/cirrhosis

**DOI:** 10.1186/s13287-024-03817-x

**Published:** 2024-07-06

**Authors:** Deyu Hu, Jiejuan Lai, Quanyu Chen, Lianhua Bai

**Affiliations:** 1 Hepatobiliary Institute, Southwest Hospital, Army Medical University, No. 30 Gaotanyan, ShapingBa Distract, Chongqing, 400038 P.R. China; 2 Bioengineering College, Chongqing University, No. 175 Gaotan, ShapingBa Distract, Chongqing, 400044 China

**Keywords:** Bone marrow-derived mesenchymal stem cells, Liver cirrhosis, Bile duct cells, Diethylnitrosamine, Tissue repair, Regeneration

## Abstract

**Background:**

Bone marrow-derived mesenchymal stem cell (_BM_MSC)-based therapy has become a major focus for treating liver fibrosis/cirrhosis. However, although these cell therapies promote the treatment of this disease, the heterogeneity of _BM_MSCs, which causes insufficient efficacy during clinical trials, has not been addressed. In this study, we describe a novel Percoll–Plate–Wait procedure (PPWP) for the isolation of an active cell subset from _BM_MSC cultures that was characterized by the expression of neuroglial antigen 2 (NG2/_BM_MSCs).

**Methods:**

By using the key method of PPWP and other classical biological techniques we compared NG2/_BM_MSCs with parental _BM_MSCs in biological and functional characteristics within a well-defined diethylnitrosamine (DEN)-induced liver fibrosis/cirrhosis injury male C57BL/6 mouse model also in a culture system. Of note, the pathological alterations in the model is quite similar to humans’.

**Results:**

The NG2/_BM_MSCs *revealed more advantages compared to parental*_*BM*_*MSC*s. They exhibited greater proliferation potential than parental _BM_MSCs, as indicated by Ki-67 immunofluorescence (IF) staining. Moreover, higher expression of SSEA-3 (a marker specific for embryonic stem cells) was detected in NG2/_BM_MSCs than in parental _BM_MSCs, which suggested that the “stemness” of NG2/_BM_MSCs was greater than that of parental _BM_MSCs. In vivo studies revealed that an injection of NG2/_BM_MSCs into mice with ongoing DEN-induced liver fibrotic/cirrhotic injury enhanced repair and functional recovery to a greater extent than in mice treated with parental _BM_MSCs. These effects were associated with the ability of NG2/_BM_MSCs to differentiate into bile duct cells (BDCs). In particular, we discovered for the first time that NG2/_BM_MSCs exhibit unique characteristics that differ from those of parental _BM_MSCs in terms of producing liver sinusoidal endothelial cells (LSECs) to reconstruct injured blood vessels and sinusoidal structures in the diseased livers, which are important for initiating hepatocyte regeneration. This unique potential may also suggest that NG2/_BM_MSCs could be an novel off-liver progenitor of LSECs. Ex vivo studies revealed that the NG2/_BM_MSCs exhibited a similar trend to that of their in vivo in terms of functional differentiation responding to the DEN-diseased injured liver cues. Additionally, the obvious core role of NG2/_BM_MSCs in supporting the functions of _BM_MSCs in bile duct repair and BDC-mediated hepatocyte regeneration might also be a novel finding.

**Conclusions:**

Overall, the PPWP-isolated NG2/_BM_MSCs could be a novel effective cell subset with increased purity to serve as a new therapeutic tool for enhancing treatment efficacy of _BM_MSCs and special seed cell source (BDCs, LSECs) also for bioliver engineering.

**Supplementary Information:**

The online version contains supplementary material available at 10.1186/s13287-024-03817-x.

## Background

Mesenchymal stem cells (MSCs) are being harnessed to develop a broad range of cellular therapies for damaged tissues [1]. MSCs can be isolated from multiple organ tissues, such as bone marrow [2], adipose tissue [3], umbilical cord blood [4] etc., although in lower numbers, while similar functional benefits have been observed. However, a major challenge in realizing the therapeutic potential of these cells in clinical trails is their heterogeneity, which limits their efficacy. Heterogeneity refers to the existence of difference cell substs with different immunophenotypes in _BM_MSC cultures. It may arise from distinct phenotypes in vivo, adaptation to ex vivo cultivation, or senescence upon expansion [5]. Therefore, a more rapid selection method for effective cell subset is warranted for the development of MSC therapies, and an immunophenotypic characterization of MSC populations is urgently needed for high-throughput enrichment of MSC progenitors.

Attempting to further transform heterogeneous MSCs into specific progenitor subsets have achieved only partial success [6], several studies have raised issues about the most effective subpopulation of cells within MSCs and defined their mode of action. O’Connor et al. [7] described a active subset with coexpression of the neuron-glial antigen 2 (NG2) immunophenotype and CD146 within human marrow-derived MSCs (h-_BM_MSCs), a well-accepted adult stem cell population in the field that is currently the most commonly used in clinical trials, but O’Connor’s study was limited by the ex vitro expansion method. We have previously described the functional benefits of ex vivo-expanded NG2-expressing cell subsets in the liver and documented their ability to promote diseased liver endogenous repair functional recovery through the direct development of specific cells from the PPWP-isolated NG2^+^ cells after transplantation into a mouse model of orally administered diethylnitrosamine (DEN)-induced liver fibrosis/cirrhosis injury [8, 9].

*This orally administered DEN mouse model is a well-defined animal model and widely used in field* [9, 10]. *The characteristics of the model include three typical pathological alterations: liver fibrosis, cirrhosis and cancer* [10], *and these changes resulting in liver damage are quite similar to those in humans* [9]. *The pathological mechanism of this model is associated with liver damage accompanied by mononuclear cell infiltration in the fibrotic phase, a condition that involves remodeling and expansion of the liver extracellular matrix (ECM)* [10]; *in the cirrhotic phase of the model, the pathological mechanism is accompanied by widespread architectural injury in the liver, a condition in which regenerative fibrotic nodules are formed to replace the normal functional liver parenchyma, remodel the vasculature and ultimately compromise liver function* [11–14].

One hypothesis that has attracted considerable attention is that MSCs are found in all tissues and that they exhibit pericyte (PC) properties [15]. Consistent with this hypothesis, both MSCs and PCs exhibit numerous stem cell properties [16, 17]. One feature shared by PCs is the expression of the cell surface glycoprotein NG2 [18], which was originally defined by antibodies directed against surface proteins in a rat cell line with glial and neuronal properties [19]. These NG2^+^ MSC-like cells sourced from other biological systems, such as liver and central nervous system (CNS) are found being able to generate functional cells for tissue repair [20, 21]. Notably, although NG2^+^ cells are present in human bone marrow MSCs (h-NG2/_BM_MSCs) like O’Connor et al. described and have a greater proliferation capacity than parental human bone marrow MSCs (h-_BM_MSCs) [7], researchers have not yet addressed whether the h-NG2/_BM_MSCs are superior to those of parental h-_BM_MSCs in biological and functional characteristics, and whether this h-NG2/_BM_MSC subset possesses advantages over parental h-_BM_MSCs in therapeuitc effect on liver fibrosis/ciurrhosis injury after their transplantation.

Given that NG2^+^ cells exist in multiple adult tissue MSCs, including _BM_MSCs, and that these cells have stem cell-like properties [22] and promote functional recovery in disease models [21], we in the present study expand upon preliminary data from our laboratory using the Percoll–Plate–Wait procedure (PPWP), which has been stably used for the isolation of NG2^+^ cells from multiple adult tissues [21, 23] to enrich NG2^+^ cell subset from heterogeneous _BM_MSC cultures (NG2/_BM_MSCs). By using the DEN mouse model, we compare and evaluate whether the ex vivo-expanded NG2/_BM_MSCs have advantages over parental _BM_MSCs in terms of biological and functional features, and therapeutic effect, for example, proliferation, injured biliary tree repair and hepatocyte regeneration etc. Importantly, we for the first time show a possibility that NG2/_BM_MSCs being as novel off-liver progenitor of LSECs for supporting injured liver repair and regeneration. Thus, this study demonstrate new advances that the PPWP-isolated NG2/_BM_MSC cell subset from both mice (m-_BM_MSCs, m-NG2/_BM_MSCs) and humans could be as novel therapeutic tool to enhance the efficacy of treatment in recipients with liver fibrosis/cirrhosis.

## Methods

### Animals

*The six- to ten-week-old C57BL/6* male healthy *mice*, 22–24 g weight, *originally* source *Jackson Laboratory USA, were used in this study.* Mice with DEN-induced liver fibrosis/cirrhosis injury were approved by the Institutional Animal Care and Use Committee of Army Medical University, Chongqing, China (No. #SYXK-PLA-2012-00120031). Animals were maintained in an air-conditioned animal center under specific pathogen-free conditions with a 12-h cycle of daylight, and unlimited access to food and water was provided to prevent dehydration.

### DEN-induced liver fibrotic/cirrhotic injury mouse model and study design

Using chemical reagent DEN to establish liver fibrotic/cirrhotic mouse model according to published methods [9, 10]. Briefly, male mice drank water containing 0.014% (0.13 mg/mL, 25.86 mg/kg) DEN daily for consecutive 10 weeks, and control animals were provided normal water. At 6 to 7 weeks of DEN administration (post-DEN), the mice were randomly assigned to two test groups: (1) DEN plus NG2/_BM_MSCs, *n* = 15; (2) DEN plus _BM_MSCs group, *n* = 15; and compared each other. The (1) and (2) were also compared with two control groups: DEN plus PBS only (3), *n* = 10 and naive group (4), *n* = 10. The mortality rate of the model was approximately 10–15%. The cells used for treatment were labeled with the fluorescent dye, CFSE (carboxyfluorescein diacetate succinimidyl ester) prior to transplantation to distinguish from host cells. Cells were injected *via* the tail vein (1 × 10^6^ cells in 200 µL per mouse) at 6 to 7 weeks post-DEN when the activation of endogenous hepatic stem cells decreased [10]. The same volume of phosphate-buffered saline (PBS) was administered (200 µL per mouse). Both cell groups received DEN continuously and were monitored in an additional four weeks. The examiners who assessed liver fibrosis/cirrhosis were not blinded to group allocation, but subsequent assessments were performed by examiners blinded to the groups.

### The PPWP approach for the isolation of NG2^+^ cells from _BM_MSC cultures

*Using the PPWP approach to isolate NG2*^*+*^*cells within*_*BM*_*MSC cultures.* The _BM_MSCs were cultured as previously reported [24]. Briefly, the C57BL/6 mice (6 mice/time) were euthanized in CO_2_ euthanasia chambers, their tibias and femurs were dissected, and all surrounding soft tissues were removed. Marrow was slowly flushed from the bones, and mononuclear cells were fractionated by gradient density centrifugation. After being washed, the fractionated cells were plated in 100-cm^2^ culture dishes, suspended in bGJb medium supplemented with 10% fetal calf serum (FCS), 100 U/mL penicillin and 100 mg/mL streptomycin and incubated in a humidified atmosphere of 37 °C and 5% CO_2_ for primary culture for ∼ 7–10 days until P2. Subsequently, the PPWP approach was used for NG2^+^ cell isolation from passage 2 (P2) _BM_MSC cultures based on this procedure (Suppl. Fig. S1Aa-d). The cells were isolated in three main steps. For each isolation, 1 × 10^6^_BM_MSCs were harvested before the cells were layered onto a Percoll gradient. The gradient was prepared as stock isotonic Percoll (SIP) at 70% purity in 1× PBS and 30% purity in 1× MEM (red), all without Ca^2+^ or Mg^2+^ ions. The m-_BM_MSCs were mixed in 7.5 mL of 30% SIP and slowly layered on top of the 70% SIP (7.5 mL). After 30 min of centrifugation at 2,000 rpm, the lipid layer on the top was carefully removed, and the interface portion (∼ 2–3 mL) was collected and plated into a conical tube. After centrifugation at 800 − 100 rpm for 5 min, the cells were suspended in 3 mL of DMEM/F12 supplemented with 10% FCS (complete medium), slowly plated in PLL-coated 75-cm^2^ flasks and maintained in an incubator at 37 °C with 5% CO_2_ for 15–20 min. Then, 10 mL of complete medium was added to 25 mL and incubated for 7–10 days. Steps (1)-(3) were repeated two or three times; the cultures that developed colonies (assumed to be NG2^+^ cells) reached 85% confluence after approximately 3–5 days and were ready for use. Cell purity was determined using an antibody against NG2 and cells for immunocytochemical analyses were seeded onto poly-l-lysine (PLL)-coated coverslips and grown for 1–3 days unless indicated otherwise. For humans, the marrow was aspirated from donors, and h-_BM_MSCs were grown in DMEM supplemented with 10% FBS. The same approach used for mice was also used for humans. The isolated NG2^+^ cells from _BM_MSC cultures were identified by Flow Cytometry (FCM) and immunofluorescence (IF) staining of antibody to NG2 antigen.

### Antibodies

Using antibodies to label specific markers for identification purpose factors. Primary antibodies with IF staining included an polyclonal rabbit antibody against anti-NG2 chondroitin sulfate proteoglycans and monoclonal mouse antibodies against stage-specific embryonic antigen 3 (SSEA-3), Ki-67, CK19, CD31, von Willebrand factor (vWF), lymphatic vessel endothelial hyaluronan receptor-1 (Lyve-1), a marker of differentiated liver sinusoidal endothelial cells (LSECs) [25], and alpha-smooth muscle actin (ɑ-SMA), a marker of activated hepatic stellate cells (HSCs). Primary antibodies for FCM included antibodies against NG2, CD9, CD73, CD105, platelet-derived growth factor receptor-beta (PDGFR-β), CD31, CD34 and CD45. Alexa Fluor 488- or Alexa Fluor 594-conjugated secondary antibodies against rabbit or mouse IgG were used.

### FCM assay

Using FCM assay to label surface proteins. Cells for immunolabeling were obtained from subconfluent cultures by two independent experiments, blinded examiners unless noted otherwise. Cell suspensions of 1 × 10^6^ cells/mL in PBS were immunolabeled in 100–500 µL aliquots with fluorochrome-conjugated, anti-mouse monoclonal antibodies at the saturating concentrations recommended by the manufacturer for 30 min on ice in the dark; the results were confirmed by titration. After 3 washes with PBS, the cells were resuspended in PBS, incubated on ice and analyzed using an Epics FC500 flow cytometer (Beckman-Coulter, Moflo, CA) with *Flow Jo software version 10.* Matched isotype controls were prepared in parallel at the same concentration of each antibody. Cell samples were analyzed and sorted by gating on live cells through forward and side scatter. All analyses were repeated at least three times, and viability was confirmed in parallel by performing Annexin V/PI staining and was routinely greater than 90%.

### Immunohistochemistry (IHC) and immunofluorescence (IF) staining

*Using IHC staining for detection of inflammation and collagen fibbers respectively. Liver tissues were fixed 10% neutral buffered formalin for 24 h followed in alcohol then xylene for paraffin embedding, cut into 5-µm sections and stained with H&E and MT. A semi-quantitative scoring was performed by a pathologist blinded to different study groups based on a standard Non-alcoholic fatty liver disease (NAFLD) Activity Score (NAS) or criteria* [26] *which commonly used in preclinical animal models and in patients* [27, 28] *from H&E staining. Fibrosis (or collagen accumulation) score was assessed systemically with pattern recognition from MT staining. Three representative areas per liver were examined and the scores of each parameter from individual animal were averaged*. *The liver samples were from at least six animals per type of staining.* IF staining for evaluation of expressive proteins in liver tissues. For example, SSEA-3, collagen-1α (Col-1α) for fibrotic load [29], Ki-67 for proliferation and ɑ-SMA for HSC activity [30]. Animals were perfused with 4% paraformaldehyde, and selected tissues were cryoprotected in 30% sucrose overnight, the livers were (a) snap frozen at -80 °C in optimal cutting temperature (OCT) mounting medium and sectioned (5-µm) on a Leica cryostat, (b) examined using H&E and MT staining and analyzed with Image J V2 software.

IF staining for evaluation of purpose proteins. Cells *from triplicate sets* were cultured on coverslips and at least three indepentdent experiments. The coverslips were fixed with 5% acid methanol (− 20 °C) for 12 min and then washed 2× for 5 min with room temperature DMEM supplemented with 5% normal goat serum (NGS). The coverslips were incubated with antibodies (diluted 1:100 in DMEM) for 25 min at 37 °C in a humidified chamber, washed 6× for 5 min with DMEM supplemented with 5% NGS, incubated with fluorescent dye-conjugated secondary antibodies diluted 1:200 in DMEM supplemented with 5% NGS for 30 min at 37 °C, washed briefly and mounted using Vectashield with 4’-6-diamidino-2-phenylindole (DAPI.) NG2^+^ cells were double-labeled with NG2 and Ki-67 antibodies for the proliferation assay, with NG2 and CK19 antibodies for identification of bile duct cells (BDC) or cholangiocytes, with NG2 and CD31, vWF for endothelial cells (ECs) [31], with LYVE1 for LSECs [32] and *with Albumin (Alb) and the glucose-6-phosphatase catalytic (G6Pc) for hepatocytes*. Each experiment was repeated at least 3 times, and the data were collected from duplicate coverslips. Proliferation and differentiation were assessed by quantifying the number of Ki67- or CK19- or LYVE-1-positive cells as a proportion of the total number of NG2- or CK19-positive cells, *For tissues, proliferation was assessed by quantifying the number of purpose marker-positive cells as a proportion of the total number of donor- or DAPI-positive cells. For cultures experiment was repeated at least 3 times with data from duplicate coverslips*. All liver sections and stained cells on a Leica cryostat (Leica CM1950, Wetzlar, Germany) were imaged with an Olympus microscope (BX53F2).

### CCK-8 assay

*Using the CCK-8 Kit to compare the growth rate between NG2/*_*BM*_*MSCs and*_*BM*_*MSCs.* Cells were cultured as triplicates and at least three times of culture sets, under their own normal conditions at the same density (1 × 10^3^ cells) in a 96-well plate (300 µL/well) at 37 °C with 5% CO_2_ for different durations. Then, 2-(2-methoxy-4-nitrophenyl)-3-(4-nitrophenyl)-5-(2,4-sulfophenyl)-2*H*tetrazole monosodium salt (WST-8) [33] was added to the plate (10 µL/well) once every 24 h and detected cell growth using a Varioskan Flash Spectral Scanning Multimode microplate reader (Thermo Scientific, Massachusetts, USA) at an optical density (OD) of 450 nm, and the cytokinesis of the cells was analyzed using Skanlt RE Varioskan Flash 2.4.3.

### Quantitative real-time reverse transcription–polymerase chain reaction (RT–qPCR)

*Using RT–qPCR assay to detect gene expression (mRNA) of purpose iteams.* RNA extraction (*n* = 6) was performed using a HiPure Total RNA Plus Mini Kit. Eight hundred nanograms of total RNA was transcribed into cDNA using the Prime Script RT Reagent Kit. The cDNA expression of specific genes was quantified with TB Green Premix Ex Tag (TaKaRa) and assessed using a quantitative PCR system (CFX96™ Real-Time System, Bio-Rad, Hercules, CA, USA). Relative gene expression was determined using the 2^−ΔΔCt^ method and analyzed using the accompanying software *(Bio-Rad CFX Maestro)*. Specific primers targeting differentiation-related genes were designed by Primer Express software (Applied Biosystems). The conditions for qRT‒PCR were as follows: 50 °C for 2 min; 95 °C for 10 min for 1 cycle, followed by 45 cycles of 60 °C for 1 min and 95 °C for 15 s [34]. All samples were normalized to the level of *actb/β actin*. The primer pairs used for RT‒qPCR are listed in Table 1.

**Table 1:** Primer pairs used for the RT‒qPCR determination

Genes	Sense (5’-3’)	Antisense (5’-3’)
*krt7/ck7*	AGGAGATCAACCGACGCAC	GTCTCGTGAAGGGTCTTGAGG
*krt19/ck19*	GGGGGTTCAGTACGCATTGG	GAGGACGAGGTCACGAAGC
*acta2/α-sma*	GTCCCAGACATCAGGGAGTAA	TCGGATACTTCAGCGTCAGGA
*lyve1*	CAGCACACTAGCCTGGTGTTA	CGCCCATGATTCTGCATGTAGA
*actb/β actin*	GGCTGTATTCCCCTCCATCG	CCAGTTGGTAACAATGCCATGT

*krt7*: keratin 7; *krt19*: keratin 19; *acta2*: actin alpha 2; *lyve1*: lymphatic vessel endothelial hyaluronan receptor 1; *actb*: actin, beta

### Serum analysis for functional hepatic proteins

Using blood samples to detect hepatic proteins in subgroups. Serum samples were from 6 animal bloods which were obtained for measurements of the levels of proteins related to hepatic function, at which time the experiment was terminated. Mice were anesthetized with isoflurane, and peripheral blood was collected from the retro-orbital plexus using a glass capillary. The samples were centrifuged at 10,000 rpm for 10 min to obtain blood serum, which was subsequently aliquoted and stored at -80 °C. The levels of *total bilirubin (TBIL), direct bilirubin (DBiL), indirect bilirubin (iBiL), alkaline phosphatase (ALP), alanine transaminases (ALT), aspartate transaminases (AST), low-density lipoprotein (LDL) and albumin (Alb) after mouse cell treatment (m-NG2/*_*BM*_*MSCs and m-*_*BM*_*MSCs)* were analyzed with a Beckman Count Chemistry Analyzer (AU5800, *DM2*) *after cell transplantation.*

### Preparation of DEN injured liver-conditioned media (_DEN_CM)

*Designed cultures in conditioned media (CM) is aiming to identifiey functional differentiation being response to injured liver cues*. The DEN-liver tissues (at 6–7 weeks post-DEN) were random collected (*n* = 3) homogenized in 1 mL of DMEM/F12 as a stock solution, pooled and stored at − 20 °C prior to use. For differentiation, grown P2 cells (NG2/_BM_MSCs, _BM_MSCs) on coverslips with the same sample were allowed to expand either in _DEN_CM or in control-conditioned medium (Ctrl-CM) for 1–6 h, after which the proportions of cells expressing the mature cholangiocyte marker CK19, EC markers of CD31 and von Willebrand factor (vWf), and the sinusoidal cell marker Lymphatic vessel endothelial hyaluronan receptor-1 (Lyve-1) were assayed. The stock solution was pooled and stored at − 20 °C prior to use and diluted at a 1:8 ratio with serum-free DMEM/F12.

All the experiments were conducted in a blinded manner. The first investigator (NP) was the only person aware of the treatment group allocation. A second investigator (OC and RNS) was responsible for conducting the TANES and functional outcome assessments, whereas a third investigator (AHS, GH, or RS) performed the data collection and tissue analysis. Finally, a fourth investigator (AMP) (also unaware of treatment) assessed, analyzed, and interpreted all the data. *The methods used in this study assessed outcome data met the assumptions of the statistical approach and the work has been reported in line with the arrive guideline 2.0*.

All the information on the primary and secondary antibodies, reagents and kits used are listed in Table 2.

**Table 2:** Antibodies (abs), reagents, and kits

Item	Antibody	Cat. No.	Application(s)	Company	City, State	Country
Primary Abs	Rabbit Polyclonal Anti-NG2	AB5320B	Immunofluorescence, Western blot analysis	Millipore	Massachusetts	USA
	Rabbit Polyclonal Anti-SSEA-3	bs-3575R		Bioss	Beijing	China
	Rabbit Polyclonal Anti-MDR1	bs-0563R				
	Rabbit Polyclonal Anti-Ki-67	bs-2130R				
	Mouse Monoclonal Anti-CK19	sc-374,192		Santa Cruz Biotechnology	California	USA
	Mouse Monoclonal Anti-α-SMA	sc-130,617				
	Mouse Monoclonal Anti-VWF	sc-365,712				
	Mouse Monoclonal Anti-CD31	102,401		Biolegend	California	USA
	Rabbit Polyclonal Anti-ZO-1	21773-1-AP		Proteintech	Wuhan	China
	Rabbit Polyclonal Anti-CFTR	20738-1-AP				
	Rabbit Polyclonal Anti-E-cadherin	20874-1-AP				
	Rabbit Polyclonal Anti-Beta catenin	17565-1-AP				
	Rabbit Polyclonal Anti-AQP1	20333-1-AP				
	Rabbit Polyclonal Anti- Collagen Type I	14695-1-AP				
	LYVE1	14-0443-80		Invitrogen	California	USA
Secondary antibodies	Cy3 AffiniPure Goat Anti-Rabbit IgG (H + L)	111-165-003	Immunofluorescence	Jackson ImmunoResearch	Pennsylvania	USA
	Alexa Fluor 488 AffiniPure Goat Anti-Rabbit IgG (H + L)	111-545-003				
	Alexa Fluor 594 AffiniPure Donkey Anti-Mouse IgG (H + L)	715-585-150				
	Cy3 AffiniPure Goat Anti-Mouse IgG (H + L)	115-165-003				
	Alexa Fluor 488 AffiniPure Goat Anti-Mouse IgG (H + L)	115-545-003				
Antibodies for FCM	Alexa Fluor 488-Mouse-NG2	562,413	Flow Cytometry (FCM)	BD Biosciences	California	USA
	FITC-Mouse-CD9	124,808		Biolegend	California	USA
	PE-Mouse-PDGFR-β	136,005				
	APC-Mouse-CD105	120,414				
	PE-Mouse-CD31	102,407				
	PE Rat IgG2a, κ Isotype Ctrl Antibody	400,507				
	FITC-Human-CD34	AH0134225		Precision BioMedicals	Chongqing	China
	PE-Human-CD73	AH0273125				
Reagents	bGJb medium (Fitton–Jackson Modification)	2,216,413	Cell culture	Gibco	California	USA
	DMEM/F-12 (1:1) basic (1X)	C11330500BT				
	Percoll	LS-0073		LANGTIAN	Chongqing	China
	Poly- L -lysine	25988-63-0	Cell culture	Sigma‒Aldrich	Missouri	USA
	DEN	N0756	Animal models			
	DAPI	C0065	Immunofluorescence	Solarbio	Beijing	China
	CFSE	1,948,067	Cell tracking	Invitrogen	California	USA
	Cell Tracker™ Red CM-DiI	C7000	Cell tracking	Invitrogen	NY	USA
	Streptomycin	C0222	Cell cultures	Beyotime,	Shanghai	China
Kits	PrimeScript™ RT reagent Kit with gDNA Eraser	RR047A	RT‒qPCR	TaKaRa Bio, Inc.	Shiga	Japan
	TB Green^®^ Premix Ex Taq™ II	RR820A				
	One Step TUNEL Apoptosis Assay Kit (Green Fluorescence)	C1086	Cell apoptosis assay	Beyotime	Shanghai	China
	Enhanced BCA Protein Assay Kit	P0010	Western blot analysis			
	Enhanced Cell Counting Kit-8	C0043	Cell proliferation assay			
	*Hematoxylin Solution*	*ZLI-9610*	*H&E*	*ZSGB-Bio*	*Beijing*	*China*
	*Eosin Dyeing Solution (alcohol-soluble)*	*BL703B*	*H&E*	*Biosharp*	*Anhui*	*China*
	*Modified Masson’s Trichrome Staining Kit*	*G1346*	*Masson*	*Solarbio*	*Beijing*	*China*

### Statistical analysis

All the data are presented as the means ± standard deviations (SDs) from at least three independent experiments. Differences between two cell groups were evaluated with a two-tailed Student’s *t* test or one-way analysis of variance (ANOVA). The results expressed as the mean ± SEM of three experiments. Statistical differences were considered significant when the *P* value was < 0.05 assessed by Student’s *t*-test according to SPSS *(version 13.0, Inc., Chicago, IL, USA)* and GraphPad Prism Versoin 8.0.2, as appropriate.

## Results

### Comparison of NG2/_BM_MSCs and parental _BM_MSCs in biological characteristics

**Fig. 1:**
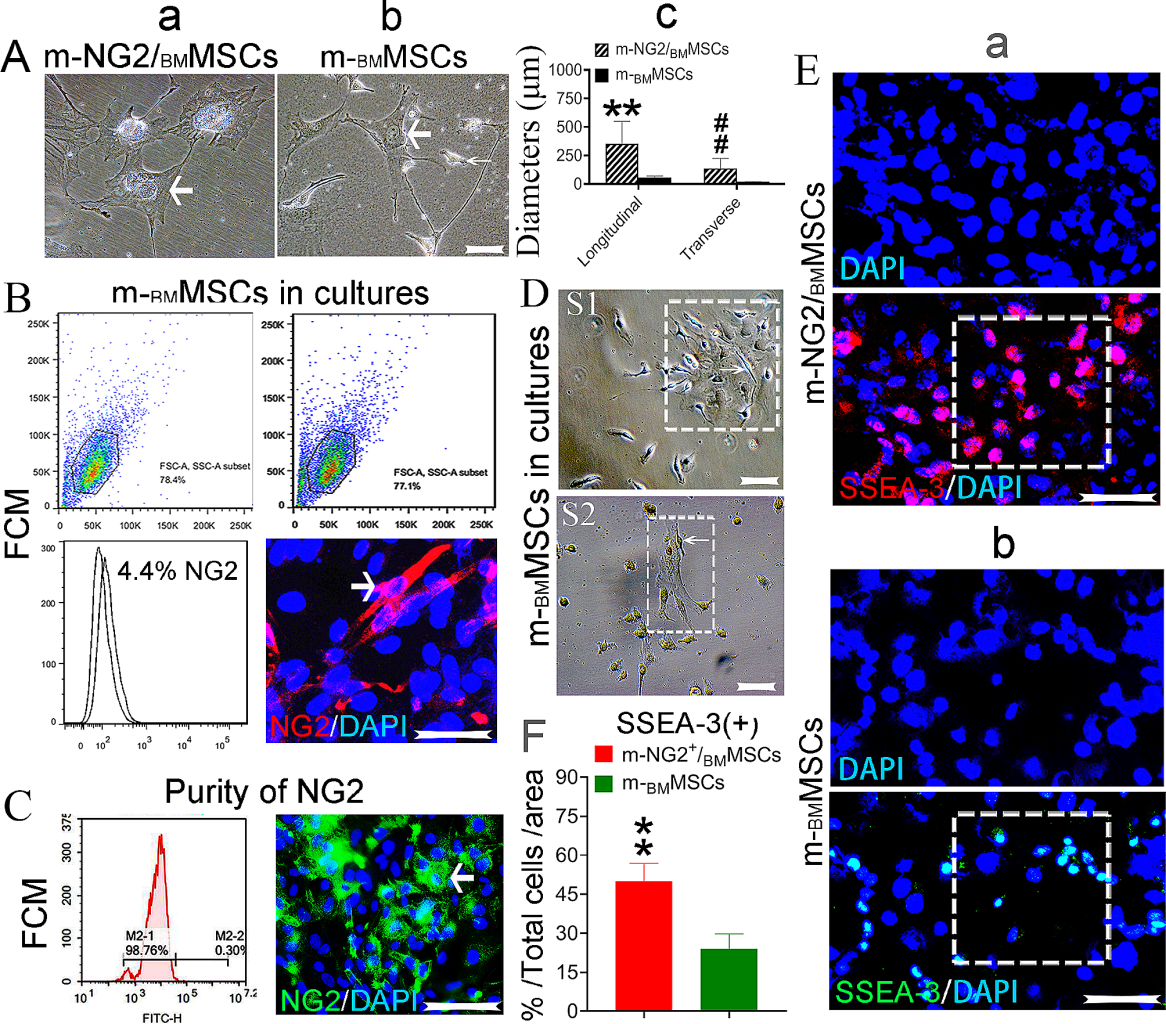
Comparison of the characteristics of ex vivo-expanded NG2/_BM_MSCs and parental_BM_MSCs. (Aa-c) Comparison of the sizes of m-NG2/_BM_MSCs **(a)** and parental m-_BM_MSCs **(b)** and quantification **(c**, *n* = 20). **(B)** FCM analyses and IF staining (red) of NG-2^+^ cells (arrow) in mouse marrow MSC (m-_BM_MSC) cultures; *n* = 6/type of experiment. **(C)** Measurement of the purity of the isolated and ex vivo-expanded NG2^+^ cells using FCM (left panel) and IF staining (green, arrow); *n* = 3/method. **(D)** NG2^+^ cells (boxes) within m-_BM_MSC cultures generated smaller spindle-shaped _BM_MSCs (thin arrows, S1 and S2 represent individual cultures. **(Ea, b)** IF staining for SSEA-3 in m-NG2/_BM_MSCs **(a**, red) and m-_BM_MSCs **(b**, green) and quantification from a and b. **(F**, boxes), *n* = 3. The means ± SDs of duplicate preparations from three independent experiments are shown. Scale bars = 200 μm for all the images except for those in A and D; scale bars = 100 μm. ** ##*p* < 0.001 compared with m-_BM_MSCs

The PPWP approach for isolating NG2^+^ cells (m-NG2/_BM_MSCs) from heterogeneously cultured mouse _BM_MSCs (m-_BM_MSCs) and the corresponding experimental procedure (*Mat/Met*) are shown in Suppl. Fig. S1Ba-d. The ex-vivo-expanded m-NG2/_BM_MSCs initially exhibited a characteristic morphology with a discoid nucleus, dense cytoplasm and irregular shape with multiple processes (Fig. 1Aa, b, bold arrows), and most passaged (P) cells exhibited a diamond-shaped flaky morphology (Fig. 1Aa; Suppl. Fig. S1Bd, pink cartoon marks assumed NG2^+^ cells), similar to the cells isolated from multiple other organs [21, 23], but those morphologies were clearly different from those of spindle-shaped m-_BM_MSCs (Fig. 1Bb, thin arrow; Suppl. Fig. S1Bd with blue cartoon marked). A comparison of the two cell types from the same passage (P2) according to size revealed that the flaky m-NG2/_BM_MSCs were significantly larger than the spindle-shaped m-_BM_MSCs from both transverse and longitudinal diameters (Fig. 1Ac, *p* < 0.001). For further characterization, the cultures were labeled with an antibody against NG2 to distinguish the cells within m-_BM_MSCs, and the results were assessed using both FCM and IF staining (red). Both methods revealed that less than 5% of NG2^+^ cells were detected in m-_BM_MSC cultures (Fig. 1B, an arrow). The isolated cells were labeled with an antibody against NG2 and subjected to FCM to determine whether the PPWP method could yield substantial numbers of NG2^+^ cells, and all the cells reached 95–98% purity using the FCM and staining (green, Fig. 1B, an arrow denotes NG2^+^ cells). Interestingly, in normal culture, larger flaky m-NG2/_BM_MSCs (boxes) were likely to produce smaller spindle-shaped m-_BM_MSCs (thin arrows, Fig. 1D; S1 and S2 represent individual cultures), which may suggest that m-NG2/_BM_MSCs retain a more immature immunophenotype than parental m-_BM_MSCs. We confirmed this finding by measuring the embryonic stem cell marker SSEA-3 [35]. IF staining revealed a higher expression of SSEA-3 in m-NG2/_BM_MSCs (red, Fig. 1Ea) than in parental m-_BM_MSCs (green, Fig. 1Eb, boxes; **F**, **p < 0.001). In addition, the FCM assay revealed similar surface expression patterns of m-NG2/_BM_MSCs (Suppl. Fig. S1C) to classic m-_BM_MSCs and the ability of these cells to differentiate into osteogenic and adipogenic cells (Suppl. Fig. S1D) [8], suggesting that the isolated m-NG2/_BM_MSC subset share some features with m-_BM_MSCs. These findings indicate that the m-NG2/_BM_MSCs isolated via the PPWP exhibit similar but mainly different biological characteristics from parental m-_BM_MSCs.

### Proliferation potential of m-NG2/_BM_MSCs in cultures

**Fig. 2:**
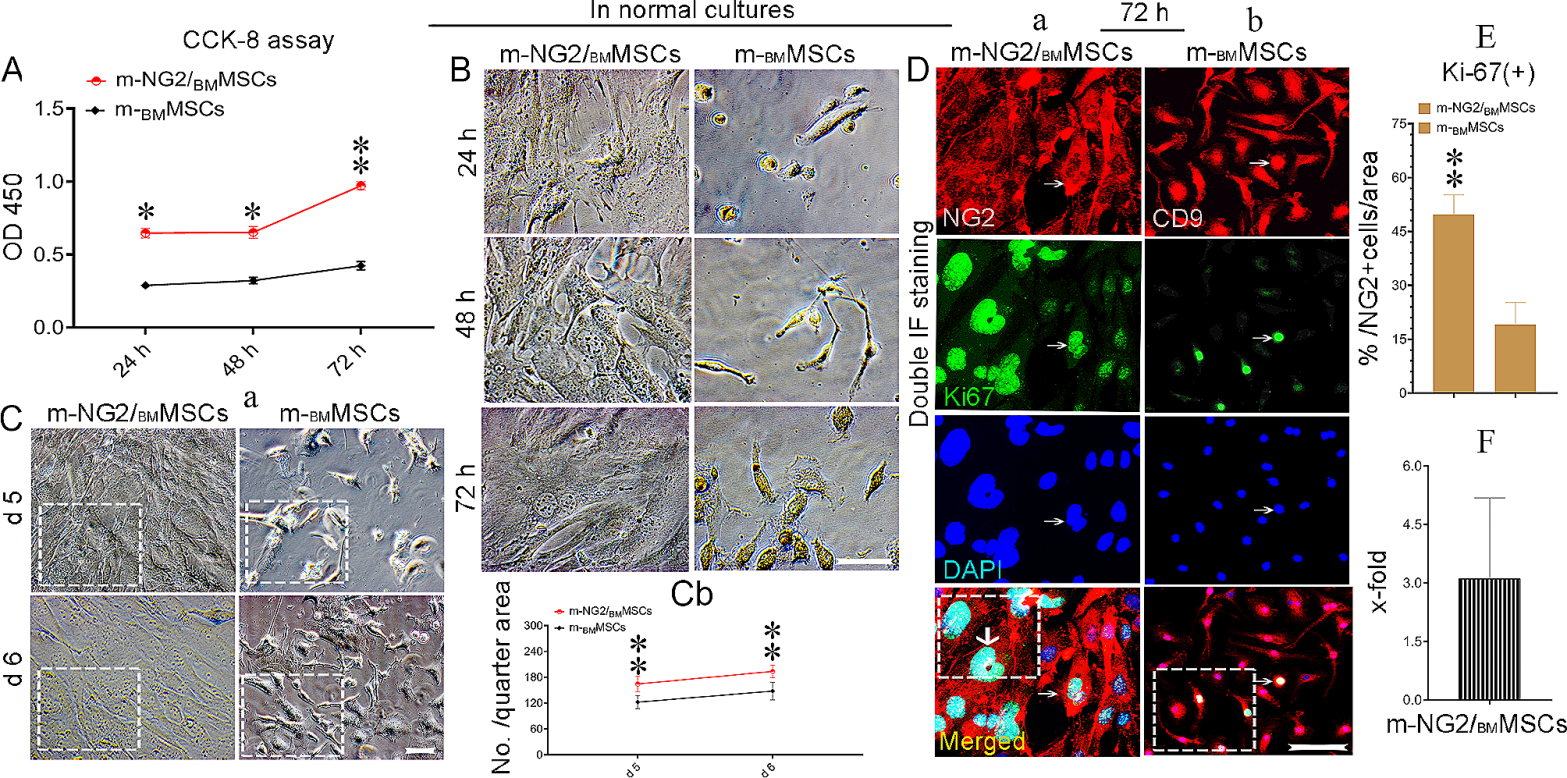
Proliferation potential of the ex vivo-expanded m-NG2/_BM_MSCs in normal cultures. (**A, B**) A CCK-8 assay for comparison of the two types of cells in growth rates from normal cultures at 24, 48, and 72 h. **(Ca, b)** Comparison of growth rates at 5 and 6 days **(a)** and quantification **(b**, boxes); *n* = 3/time point. **(Da, b)** Double IF staining to identify m-NG2/_BM_MSCs **(a**, NG2, red) costained with Ki-67 (green, **a**, arrows), and the same procedure was used for parental _BM_MSCs **(b**, CD9, red, arrows); *n* = 6. **(E)** Quantification of the percentage of Ki-67-positive cells in D (merged, boxes) after 72 h in cultures; *n* = 6. **(F)** Fold changes in the expression of Ki-67 in m-NG2/_BM_MSCs over that in _BM_MSCs were analyzed as described in (E). The means ± SDs of duplicate preparations from three independent experiments are shown. Scale bars: Ca = 100 μm, others = 200 μm. * *p* < 0.05 and **p < 0.001 compared with m-_BM_MSCs

The growth and proliferation rates were detected in normal cultures to determine whether ex vivo-expanded m-NG2/_BM_MSCs could also have active potential similar to the cells in vivo [7]. The CCK-8 assay (Fig. 2A) revealed that the growth rate of the m-NG2/_BM_MSCs cultured in normal medium (Fig. 2B) was higher than that of the parental m-_BM_MSCs at all time points (Fig. 2A, B) and was obvious after 72 h (Fig. 2A, B, bottom panels, **Ca, b**, boxes **p < 0.001). Double IF staining revealed that by 72 h, approximately 49.71%± 5.46% of the m-NG2/_BM_MSCs (NG2, red) expressed Ki-67 (green, arrows, Fig. 2Da), and this percentage was significantly higher than that of the m-_BM_MSCs (CD9, red; Fig. 2Db, 19.04%± 6.19%, **E**, boxes). An approximately 3-fold higher percentage of proliferating m-NG2/_BM_MSCs than parental m-_BM_MSCs was detected (Fig. 2F). When tracing the size of the proliferated clones, we found much larger for m-NG2/_BM_MSCs (Fig. 2D, a bold arrow) which was not observed for the m-_BM_MSCs, suggesting that the proliferative capacity of the cultured m-NG2/_BM_MSCs was superior to that of the parental m-_BM_MSCs. These findings indicate that ex vivo-expanded m-NG2/_BM_MSCs also possess proliferation potential, which may explain why the new cell subset was more immature than the parental m-_BM_MSCs in maintaining more “stemness” in culture, as indicated by self-renewal.

### Advantages of donor m-NG2/_BM_MSCs in promoting endogenous BDCs repair and functional recovery in DEN-induced liver fibrosis/cirrhosis mouse model

**Fig. 3:**
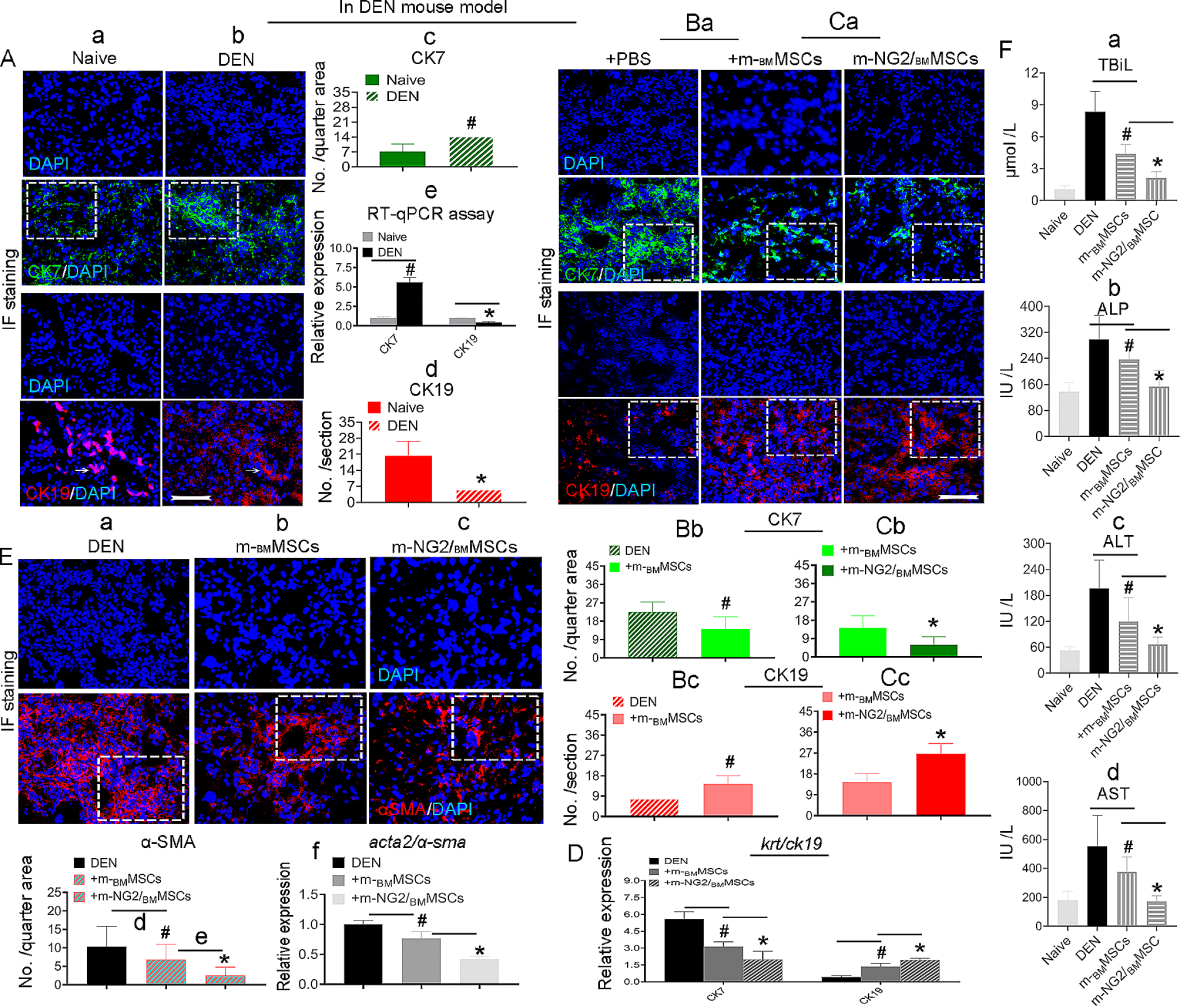
Advantages of m-NG2/_BM_MSCs over parental m-_BM_MSCs in promoting endogenous bile duct repair and improving functions in a DEN-induced mouse model four weeks after cell transplantation. (**Aa-e**) IF staining for CK7 (green) and CK19 (red) in naive **(a)** and DEN-treated **(b)** livers and quantification of CK7 **(c)** and CK19 **(d)** expression. RT‒qPCR analysis of the mRNA expression showed a similar trend **(e)**; *n* = 6/group. **(Ba-c)** IF staining **f**or CK7 (green) and CK19 (red) in DEN-treated (+ PBS) and m-_BM_MSC-treated livers **(a)** and was quantified **(b, c**, boxes), *n* = 6/group. **(Ca-c)** The same staining procedure was used for comparisons of m-_BM_MSC- and m-NG2/_BM_MSC-treated livers **(a)**, and the results were quantified **(b, c**, boxes); *n* = 6/group. **(D)** RT‒qPCR analysis of the expression of the *ck7* and *ck19* genes in the subgroups; *n* = 3. **(Ea-f)** IF staining for ɑ-SMA expression (red) in the liver according to subgroups **(a-c)** and analyses at both the protein **(d, e)** and mRNA **(f)** levels; *n* = 6/per group. **(F)** The serum levels of TBIL, ALP, ALT and AST in the subgroups were measured with a Beckman Coulter Chemistry Analyzer (*n* = 3/set). The data are presented as the means ± SDs from several independent experiments. Scale bar = 200 μm. A, *#*p* < 0.05 compared with naive; B, #*p* < 0.05 compared with DEN (+ PBS); C, *p < 0.05 compared with m-_BM_MSCs; D, #*p < 0.05 compared with DEN or m-_BM_MSCs; E, #*p < 0.05 compared with DEN or m-_BM_MSCs; F, #*p < 0.05 compared with DEN or m-_BM_MSCs

Oral administration of DEN results in bile duct damage, and m-_BM_MSCs are able to repair this damage [8]. We used antibodies against CK7 and CK19, the markers for BDCs, to determine whether m-NG2/_BM_MSCs were better than m-_BM_MSCs in this regard. Compared with naive livers (Fig. 3Aa, c, d), CK7 expression (green, boxes) increased while CK19 expression decreased Fig. 3Ab, c, d, red, arrows) at 6 weeks after DEN administration according to IF staining. RT‒qPCR revealed a similar trend (Fig. 3Ae), suggesting that the CK7- and CK19-based biliary injury during the course DEN-model can be used for further BDC repair studies. As such, we next attempted to compare repair by these two types of stem cells. Cells were infused at 6–7 weeks post-DEN when endogenous hepatic stem/progenitor cell activity decreased dramatically [8], and the animals were evaluated 4 weeks after cell transplantation. IF staining revealed that the expression of host CK7 (green, Fig. 3Ba) and CK19 (red) after treatment with PBS (left panels) was relatively constant in the DEN-induced animals (Fig. 3Ab), while in the animals that received m-_BM_MSCs (Fig. 3Ba, middle panels), considerable improvements in both CK7 (Fig. 3Bb, boxes) and CK19 (Fig. 3Bc, boxes) expression were observed. However, the mice that received m-NG2/_BM_MSCs (Fig. 3Ca, right panels) showed even greater improvements than the mice that received parental m-_BM_MSCs (Fig. 3Cb, **c**, boxes), with an approximately 1.5-fold improvement in the efficacy of m-NG2/_BM_MSCs compared with that of m-_BM_MSCs for CK17 (14.17 ± 5.99/6.12 ± 3.83) (Fig. 3Cb) and an approximately 2.3-fold improvement for CK19 (26.71 ± 4.39/14.50 ± 3.57, Fig. 3Cc). A similar trend was detected at the mRNA level using RT‒qPCR (Fig. 3D), suggesting that m-NG2/_BM_MSCs were superior to parental m-_BM_MSCs in repairing the damaged bile ducts. We used an antibody against muscle actin alpha (ɑ-SMA), a marker for activated hepatic stellate cells (HSCs) [36] to determine whether this repair could improve liver fibrotic load, and -detected a greater reduction in mice that treated with m-_BM_MSCs (Fig. 3Eb) compared to DEN-treated mice (Fig. 3Ea). However, although these differences were significant (Fig. 3Ed, #*p* < 0.05), the effect was not as strong as that of m-NG2/_BM_MSCs (Fig. 3Ec, e, *p < 0.05). A similar expression pattern was detected at the mRNA level using RT‒qPCR (Fig. 3Ef). This result is consistent with the improvements in the levels of the functional hepatic proteins TBiL, ALP, ALT and AST (Fig. 3Fa-d), *as well as inflammatory infiltration and collagen fibers, as determined using H&E*(Suppl. Fig. S2Ai) *and Masson’s trichrome (MT) staining* (Suppl. Fig. S2Bi) *thatscored with NAS criteria***(A-Bii-iv**, *Mat/Met*) [26], suggesting that improved BDC repair by m-NG2/_BM_MSCs could support improvements in systemic functions, *fibrotic load and pathological conditions*. These findings indicate that DEN-induced biliary injury can be better repaired by the novel m-NG2/_BM_MSC cell subset than by parental m-_BM_MSCs, leading to the inhibition of inflammation, alleviation of the fibrotic load and promotion of functional recovery.

### The m-NG2/_BM_MSC subset promotes BDC-mediated regeneration and plays a core role in m-_BM_MSC functions

**Fig. 4:**
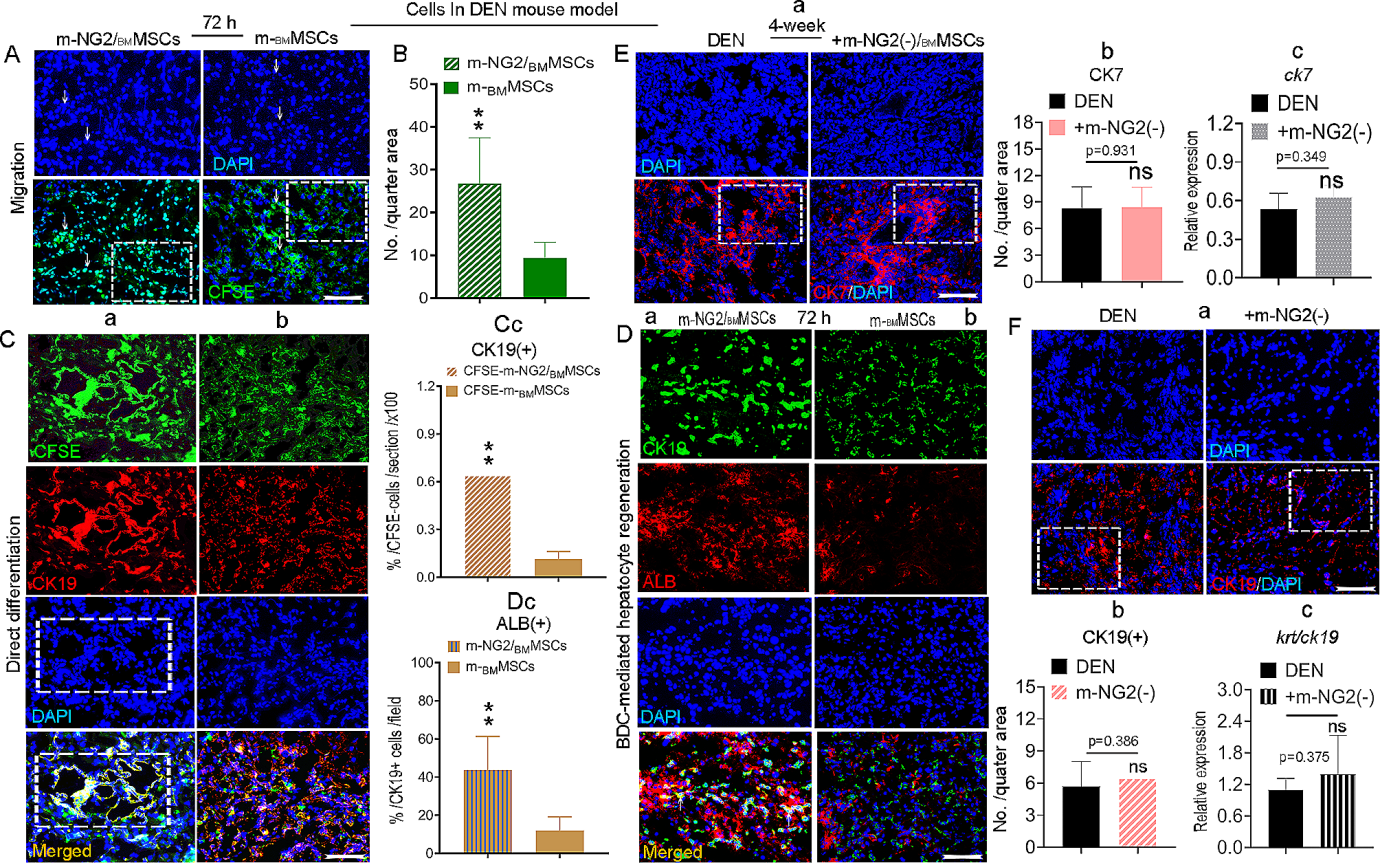
m-NG2/_BM_MSCs homed to lesions, differentiated into BDCs and promoted host BDC-mediated hepatocyte regeneration as a core component of m-_BM_MSC functions. (**A**) CFSE-labeled m-NG2/_BM_MSCs and m-_BM_MSCs migrated into injured areas of the liver 72 h after transplantation. **(B)** Quantitative analysis of the data in A (boxes, *n* = 6). **(Ca-c)** IF staining of CK19^+^ cells (red) differentiated from CFSE-labeled m-NG2/_BM_MSCs **(a)** and m-_BM_MSCs **(b)** and quantification **(c**, merged, *n* = 6, the. The boxes represent CK19^+^ cells that formed vessel-like structures). **(Da-c)** Double IF staining of host CK19^+^ cells (green, arrows) after 72 h covered by ALB^+^ cells (red) in mice (livers) treated with m-NG2/_BM_MSCs **(a)** or m-_BM_MSCs **(b)** and quantification of the merged cells (arrows, **c**, *n* = 6). **(Ea-c) A**nalysis of CK7 by IF staining (red) **(a, b)** and mRNA levels using RT‒qPCR **(c)**; *n* = 6. **(Fa-c)** Similar analysis to E for CK19, *n* = 6. The data are presented as the means ± SDs from several independent experiments. Scale bar = 200 μm. **P < 0.001 compared with m-_BM_MSCs; ns represents no significant difference

Cells were labeled with CFSE prior to injection to determine whether the improved functional efficacy of m-NG2/_BM_MSCs was associated with migration to injured liver areas. By 72 h, both cell types were detected around vessel-like structures (arrows) in the DEN-injured livers (Fig. 4A), and significantly greater numbers were detected in the m-NG2/_BM_MSC group than in the m-_BM_MSC group (Fig. 4B, boxes), suggesting that m-NG2/_BM_MSCs were more sensitive to injury signals than m-_BM_MSCs. Very few injected cells were detectable in the naive livers (not shown). Furthermore, we detected the direct differentiation potential of these CFSE-labeled cells in the injured liver via IF staining and found that a greater proportion of CK19^+^ cells (red) differentiated directly from m-NG2/_BM_MSCs (Fig. 4Ca) than from m-_BM_MSCs (Fig. 4Cb, merged). Approximately 64.05%± 0.11% of CK19^+^ cells differentiated from m-NG2/_BM_MSCs, which was a significantly higher value than the number of cells that differentiated from m-_BM_MSCs (11.54%± 0.04%; Fig. 4Cc, merged *p* < 0.001). Notably, in this study, we observed that CK19^+^ cells formed vessel-like structures from m-NG2/_BM_MSCs (Fig. 4Ca, boxes), which was not observed for parental m-_BM_MSCs (Fig. 4Cb), suggesting that m-NG2/_BM_MSCs not only were advantageous for direct differentiation into cholangiocytes but also may reconstruct bile ducts after 4 weeks of cell treatment. Interestingly, in the present study, double IF staining revealed that during the donor cell treatment period, approximately 43.67%± 7.44% of the ALB^+^ cells (red, arrows) were directly differentiated from host CK19^+^ cells (green) in mice (livers) treated with m-NG2/_BM_MSCs (Fig. 4Da, merged), but only a few of the ALB^+^ cells (< 5%) differentiated in mice treated with parental m_BM_MSCs (Fig. 4Db, merged, c), suggesting that m-NG2/_BM_MSCs could stimulate endogenous cholangiocyte-mediated hepatocyte regeneration in the diseased liver. owever, when NG2^+^ cells were removed from m-_BM_MSCs by magnetic activated cell sorting [MACS, m-NG2(-)/_BM_MSCs] [37], no improvements in biliary repair were detected; these effects were represented by CK7 (Fig. 4Ea, boxes, **b**, *p* = 0.931) or CK19 (Fig. 4**Fa**, boxes, **b**, *p* = 0.386) expression, comparable to DEN mice, and the mRNA levels were similar (CK7: Fig. 4Ec, *p* = 0.349; CK19: Fig. 4Fc, *p* = 0.375), suggesting that NG2^+^ cells play a core role in the function of _BM_MSCs in BDC-mediated regulation. These findings indicate that m-NG2/_BM_MSCs are important not only for enhancing the response to injury signals, bile duct repair and BDC-mediated hepatocyte regeneration but also for supporting the functionality of _BM_MSCs in BDC repair it associated regeneration.

### m-NG2/_BM_MSCs potentially generate cholangiocytes and endothelial cells in response to DEN-induced cues, particularly in the repair of sinusoidal structures

**Fig. 5:**
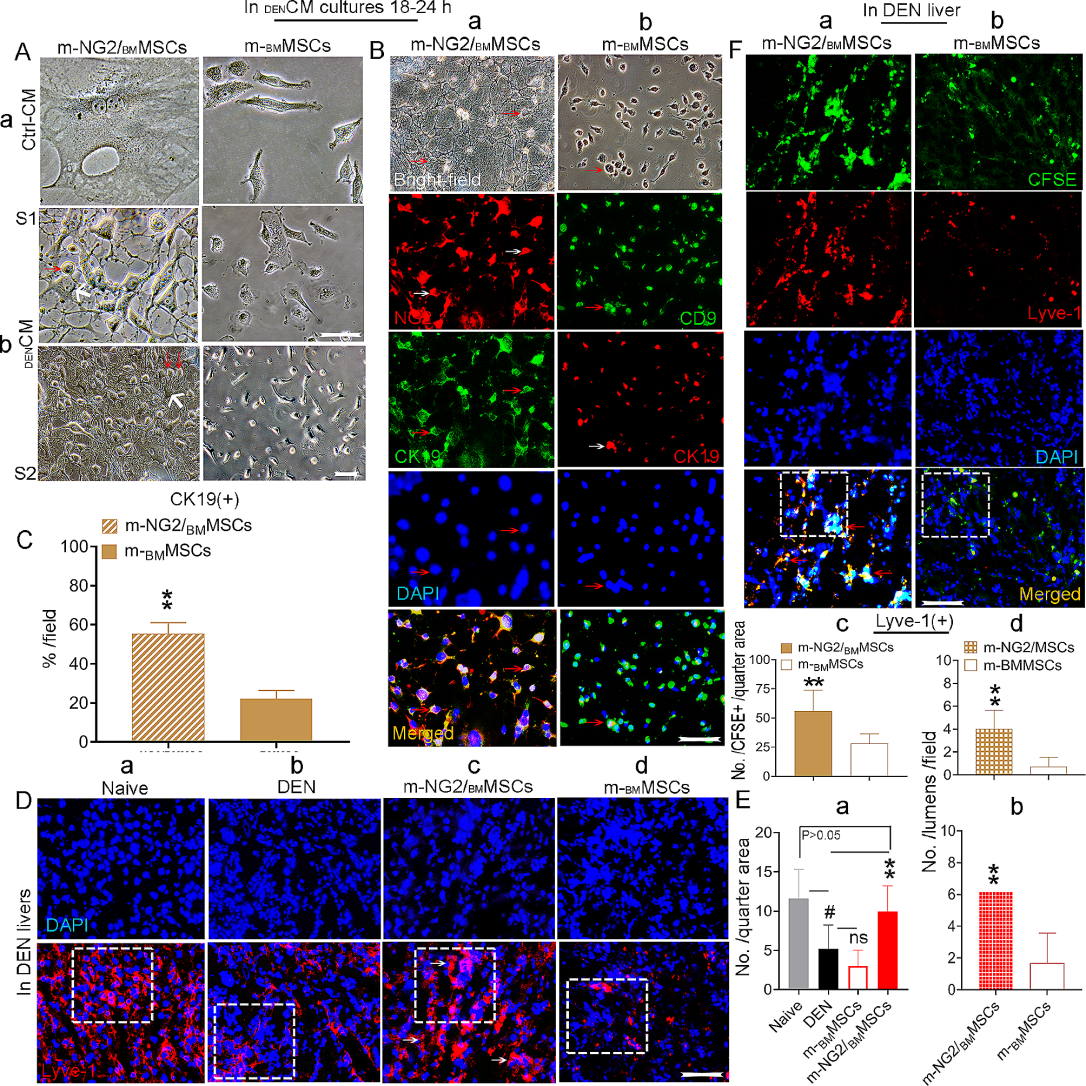
Direct differentiation of BDCs from m-NG2/_BM_MSCs in response to DEN-induced liver injury cues. (**Aa-b**) The morphology of m-NG2/_BM_MSCs in control medium (Ctrl-CM, **a)** and in the _DEN_CM **(b)**, bold arrows indicate assumed NG2^+^ cells, red arrows indicate as bile duct-like cells. Little change was observed in parental m-_BM_MSCs during this period **(b**, right panels) compared to that in Ctrl-CM **(a**, right panel): S1, S2 in b represent individuals, scale bar = 100 μm; remaining panels: scale bar = 200 μm. **(Ba, b)** Double IF staining for NG2^+^ cells (red) or CD9^+^ cells (green) covered by CK19^+^ cells (green/red) of m-NG2/_BM_MSCs **(a)** and m-_BM_MSCs **(b)** cultured in _DEN_CM for 18–24 h. **(C)** Quantification (Ba, b) of the merged cells. **(Da-d)** IF staining (red) for host Lyve-1^+^ cell expression (boxes) in subgroups of liver sections. **(Ea, b)** Quantificative analysis of Lyve-1 expression in subgroups from boxes in Da-d **(a)** and the number of vessel-like structures formed from the groups in Dc, d **(b**, *n* = 6). **(Fa-d)** Direct differentiation of Lyve-1^+^ cells from donor m-NG2/_BM_MSCs **(a)** and parental m-_BM_MSCs **(b)** in _DEN_CM and comparative quantification of total numbers per quarter area (boxes, **c)** and the number of vessel-like formations (red arrows, **d**, *n* = 6). At least three independent experiments were performed, and the data are presented as the means ± SDs. Scale bar = 200 μm. #*p* < 0.05 compared with naive; **p < 0.001 compared with m-_BM_MSCs; ns: no significance compared with m-_BM_MSCs

We added m-NG2/_BM_MSCs to conditioned media (CM) generated from DEN-induced diseased livers (_DEN_CM), and the CK19^+^ BDC lineages that developed were compared with parental m-_BM_MSCs to confirm the occurrence of advanced BDC differentiation induced by m-NG2/_BM_MSCs in DEN-treated model livers. By 18–24 h, bile duct-like cells (red arrows, Fig. 5Ab), based on morphology, were generated in large numbers from m-NG2/_BM_MSCs (white arrows, Fig. 5Ab), while m-_BM_MSCs produced fewer of these cells compared to m-NG2/_BM_MSCs (Fig. 5Ab, right panels; S1, S2 represent different scales), and < 1% of the bile duct-like cells were produced in normal culture medium (Ctrl-CM, Fig. 5Aa). Double IF staining revealed that the cells with a change in morphology were CK19-positive (third panels, Fig. 5B); approximately 55.31%± 5.75% of the m-NG2/_BM_MSCs (NG2, red, second panel) were CK19^+^ cells (green, Fig. 5B**a**, merged, arrows), and 22.21%± 4.20% of the CK19^+^ cells (red) were m-_BM_MSCs (CD9, green, second panel, Fig. 5B**b**, merged, arrows, **C)**, suggesting that both cell types tended to undergo differentiation into BDCs in response to DEN signaling, while m-NG2/_BM_MSCs exhibited more obvious changes. In these cultures, we also investigated the advantages of m-NG2/_BM_MSCs in terms of their CD31^+−^ and vWf^+^ -mediated EC differentiation potential, and we observed greater differentiation from m-NG2/_BM_MSCs (Suppl. Fig. S2Ca, Da) than from m-_BM_MSCs (Suppl. Fig. S2Cb, Db; **Cc**, **Dc**, merged, boxes). Notably, we observed greater numbers of CD31^+^ and vWf^+^ cell-mediated vessel-like structures formed by m-NG2/_BM_MSCs (Supl-fig. S2Ca, Da, arrows, E), and these phenomena were not observed for m-_BM_MSCs (Supl-fig. S2Cb, Db, F), indicating a significant difference.

Liver sinusoidal endothelial cells (LSECs) or sinusoidal cells are special ECs that are important for initiating liver regeneration [38]; thus, we speculate that the advantages of m-NG2/_BM_MSCs for promoting injured liver repair and functional recovery may also include sinusoidal contributions. We tested this possibility by using the DEN model, also a marker specific for LSECs, Lyve-1 [39]. IF staining revealed that in the animals that received m-NG2/_BM_MSCs, the host proportion of Lyve-1^+^ cells (red, Fig. 5Dc) increased by approximately 51%, which differed significantly from that in the mice with ongoing DEN (Fig. 5Db, **Ea**, boxes, **p < 0.001: red bar vs. black bar), in which the number of Lyve-1^+^ cells was dramatically decreased compared to that in the naive mice (Fig. 5Da, **Ea**, boxes, #*p* < 0.05: black bar vs. gray bar). A significant difference was not detected in the mice treated with parental m-_BM_MSCs (Fig. 5Dd) which was comparable to the DEN group (Fig. 5Ea, boxes, ns: red empty bar vs. black bar). Further analysis of these animals revealed that the proportion of Lyve-1^+^ cells (red) that directly developed from CFSE-labeled m-_BM_MSCs was approximately 28%± 8.31% (Fig. 5Fb); in contrast, after 4 weeks of treatment with the same labeled m-NG2/_BM_MSCs, the proportion of Lyve-1^+^ cells reached approximately 56% ± 18.01 (Fig. 5Fa, c, merged, boxes). Interestingly, in this experiment, many Lyve-1^+^ cells formed vessel-like structures (Fig. 5Dc, white arrows; **Fa**, red arrows), and this phenomenon was not observed for parental m-_BM_MSCs (Fig. 5Dd; Eb, Fb, d), suggesting that the unique advantages of m-NG2/_BM_MSCs in sinusoidal repair and reconstruction could also be the mechanism responsible for supporting host functional recovery. These observations indicate that m-NG2/_BM_MSCs can differentiate into BDCs and LSECs in response to fibrotic/cirrhotic liver injury signals to support regeneration and functional recovery, and the advanced, unique capacity of m-NG2/_BM_MSCs to differentiate into LSECs may also suggest that these cells may be novel off-liver progenitors of LSECs for injured sinusoidal reconstruction.

### Characterization of ex vivo-expanded NG2^+^ cells isolated from human marrow MSCs and the advantages of these cells in exerting therapeutic effects on DEN-induced liver disease

**Fig. 6:**
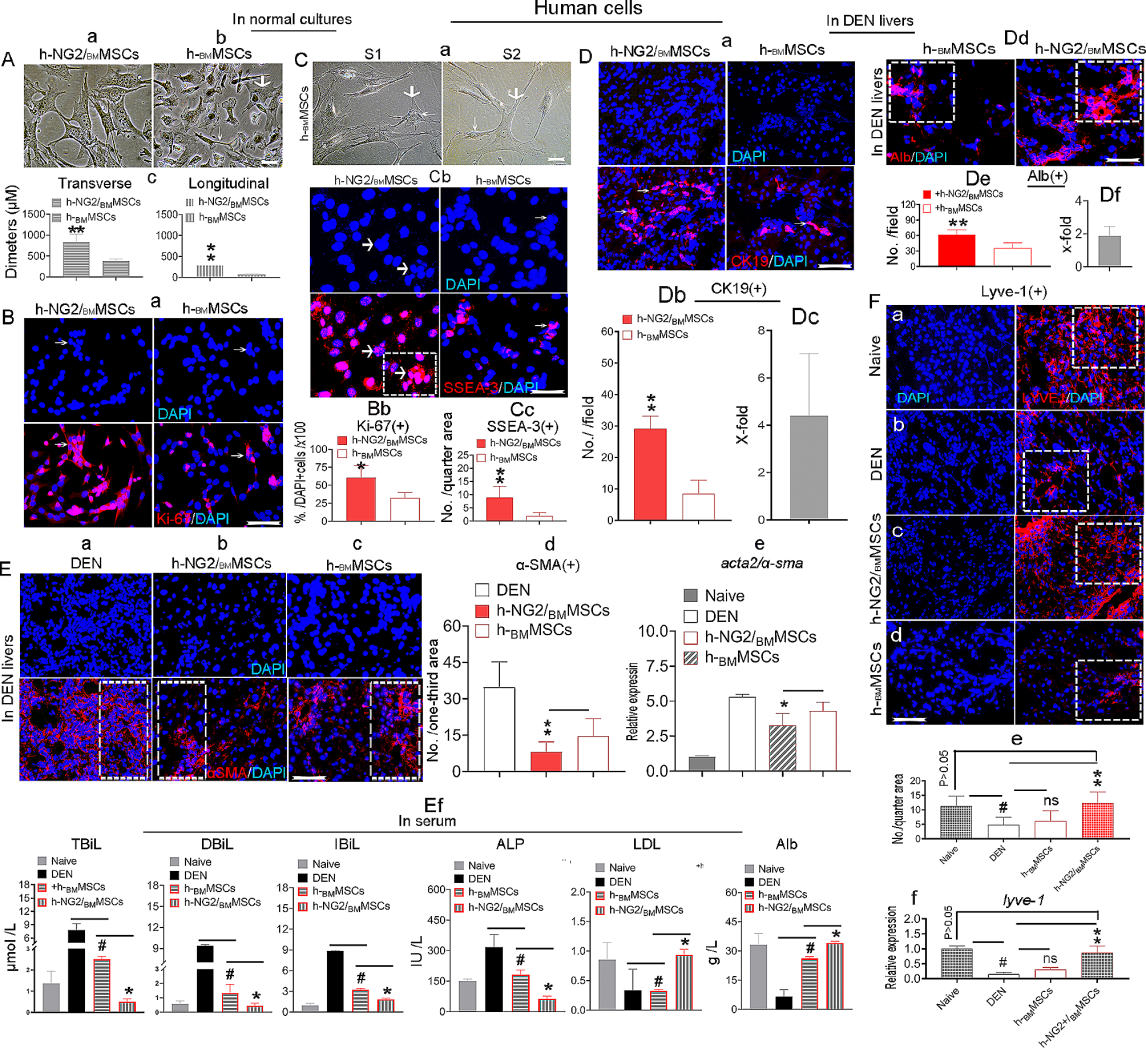
Characterization of NG2^+^ cells isolated from human marrow MSCs (h-_BM_MSCs) via the PPWP. (Aa-c) h-_BM_MSC-sourced NG2^+^ cells (h-NG2/_BM_MSCs) **(a)** isolated from h-_BM_MSCs **(b)** and comparative analysis in size **(c)**. The bold arrow indicates NG2^+^ cells, and the thin arrow indicates spindle-shaped _BM_MSCs. **(Ba, b)** IF staining for Ki-67^+^ cells (red, arrows) in the two types of cells when their in normal cultures **(a)** and quantification **(b)**; *n* = 3. **(Ca-c)** In normal _BM_MSC cultures, larger flaky cells (a bold arrow, assumed to indicate NG2^+^ cells) appeared to produce smaller spindle-shaped cells (a thin arrow) **(a**, S1 and S2 represent individual cultures). IF staining for SSEA-3 (arrows, **b)** and comparative quantification of the two types of cells are shown **(c)**; the box shows a cell clone. **(Da-f)** IF staining (red) of host CK19^+^ and Alb^+^ cells in the liver subgroups four weeks after cell transplantation **(a, d)**, quantification (**b**, arrows; **e**, boxes)*n* = 6, and x-fold changes in host CK19^+^ cells stimulated by h-NG2/_BM_MSCs compared with those stimulated by parental h-_BM_MSCs **(c, f)**. **(Ea-f)** IF staining (red) of host ɑ-SMA^+^ cells in DEN-induced mouse livers 4 weeks after transplantation of the two types of donor cells **(b, c)** compared to DEN **(a)** and quantification of protein (**d**, boxes, *n* = 6) and mRNA levels using RT‒qPCR **(e**, *n* = 6); *blood functional hepatic parameters were also compared in these subgroups***(f**, *n = 6***)**. **(Fa-f)** IF staining (red) for host Lyve-1^+^ cells in subgroups of naive (a), DEN (b), two types of donor cells (c, d) four weeks after cell transplantation, and quantification of the number of Lyve-1^+^ cells per quarter area (boxes) of the staining **(e**, *n* = 6**);** mRNA levels in livers from the same subgroups were determined using RT‒qPCR **(f**, *n* = 6). Scale bar = 200 μm in all images; at least three independent experiments were performed, and the data are presented as the means ± SDs. *#*p* < 0.05 compared with naive or DEN or h-_BM_MSCs; **p < 0.001 h-_BM_MSCs; ns: indicate no significance

Finally, we evaluated whether human marrow MSC-sourced NG2^+^ cells (h-NG2/_BM_MSCs) could also be advanced like animal cells, aiming to improv the quality and enhance the efficacy of heterogeneous human _BM_MSCs (h-_BM_MSCs). An FCM assay detected ∼ 8–10% of the assumed NG2- cells (an arrow, Suppl. Fig. S3A) in the normal h-_BM_MSC cultures (Fig. 6**Ab**, a bold arrow). Using the PPWP approach (Suppl. Fig. S1A), we successfully obtained the appropriate fraction (arrow, lower NG2^+^ cell proportion; Suppl. Fig. S3Ba) and expanded it in vitro (Fig. 6Aa; Suppl. Fig. S3Bb). The in vitro-expanded h-NG2/_BM_MSCs showed > 95% purity, as determined by both IF staining (red, an arrow, Suppl. Fig. S3Ca) and FCM (Suppl. Fig. S3Cb), suggesting that the PPWP approach is also applicable to human cells. A series of comparison studies revealed that the transverse/longitudinal diameter of h-NG2/_BM_MSCs was also significantly larger than that of parental h-_BM_MSCs (Fig. 6Aa, a thin arrow, **c**, **p < 0.001). IF staining for Ki-67 (red, arrows) revealed that the proliferation potential of the cells was greater than that of the parental h-_BM_MSCs (Fig. 6Ba, arrows, **b**), similar to what was observed in vivo [7]. In the cultures, we also observed that the larger cells (bold arrows, assumed to be NG2^+^ cells) produced smaller spindle-shaped cells (thin arrows, Fig. 6Ca, S1 and S2 represent individual cultures) that were similar to those of the animal cells described above, were more strongly labeled by SSEA-3 of h-NG2/_BM_MSCs than parental h-_BM_MSCs(arrows, Fig. 6Cb, arrow, c), and formed larger colonies (Fig. 6Cb, **a** box), supporting the greater “stemness” of h-NG2/_BM_MSCs than h-_BM_MSCs.

After the cells were infused into the DEN model at 6–7 weeks post-DEN admonstration, h-NG2/_BM_MSCs also exhibited advantages in terms of endogenous cholangiocyte regeneration and functional recovery compared with parental h-_BM_MSCs four weeks after cell transplantation. For example, the number of host CK19^+^ cholangiocytes (red) was greater (29.18 ± 3.96; Fig. 6Da; left panels) than that of the mice that received parental h-_BM_MSCs (8.50 ± 4.62; Fig. 6Da; right panels; **b**, arrows), an approximately 4.5-fold increase (Fig. 6Dc). *The significant potential of h-NG2/*_*BM*_*MSCs was also shown for host mature hepatocyte regeneration, as indicated by the presence of albumin***(***Alb*, Fig. 6Dd, e, *boxes***)***and G6Pc* (Suppl. Fig. S3Da-c, *arrows***)**, *another marker for mature hepatocytes* [40], *approximately 2-fold and 2.5-fold higher than that of parental h-*_*BM*_*MSCs, respectively* (Fig. 6Df; Suppl. Fig. S3 Dd). By analyzing host ɑ-SMA expression via IF staining (red), we found that both cell types had the capacity to reduce the number of ɑ-SMA^+^ cells (Fig. 6Eb, c) compared to that in DEN-treated mice (Fig. 6Ea, boxes) 4 weeks after cell transplantation, but the significantly greater potential of h-NG2/_BM_MSCs was exhibited at both the protein (Fig. 6Ed, red bar, **p < 0.001) and mRNA (Fig. 6Ee, *P < 0.05) levels, suggesting that h-NG2/_BM_MSC treatment improved the fibrotic load. *Further measurement of hepatic parameters in blood revealed that h-NG2/*_*BM*_*MSCs improved the serum levels of TBiL, DBiL, IBiL, ALP, LDL and Alb (*Fig. 6Ef). *This evidence could support the notion that h-NG2/*_*BM*_*MSCs have superior therapeutic effects to parental h-*_*BM*_*MSCs on liver diseases.* Interestingly, when analyzed by performing IF staining (red) of Lyve-1 expression in the host liver, we obtained evidence similar to that collected for animal cells regarding sinusoidal repair in DEN-induced livers [Fig. 6F**(a-d)].** We detected ∼ 5 ± 3.06 Lyve-1^+^ cells/per quarter area **(b)** in DEN mice, which was significantly lower than that in naive livers **(a**, ∼ 11.58 ± 3.72/quarter area/boxes; **e**, #*p* < 0.05). The number of Lyve-1^+^ cells was significantly greater in the h-NG2/_BM_MSC-treated mice than in the DEN-treated mice **(c,** ∼ 12 ± 3.72/quarter area/boxes; Fig. 6F**b, e** red bar, **p > 0.001), and these numbers were comparable to those in the naive mice (Fig. 6F**a**, **e**, gray bar, *p* > 0.05). In contrast, compared with DEN-treated mice, mice that received h-_BM_MSCs **(d)** failed to stimulate host Lyve-1^+^ cells at either the protein (Fig. 6F**e**, boxes, ns *p* > 0.05) or mRNA level, according to RT‒qPCR (Fig. 6F**f**, ns *p* > 0.05), suggesting that human-sourced h-NG2/_BM_MSCs also possessed the unique property of reconstructing damaged sinusoidal structures. In addition, as determined using FCM, the h-NG2/_BM_MSCs expressed similar patterns of surface markers (Suppl. Fig. S3E), suggesting that the isolated h-NG2/_BM_MSCs still shared some characteristics with the h-_BM_MSCs. These findings indicate that the PPWP approach is suitable for human samples and that ex vivo-expanded human _BM_MSC-sourced NG2^+^ cells may be novel off-liver precursors of BDCs, particularly LSECs, and therapeutic tools for treating patients with liver fibrotic/cirrhotic diseases.

### Potential of h-NG2/_BM_MSCs to develop BDCs and unique capacity to directly differentiate into LSECs in response to DEN-induced cues

**Fig. 7:**
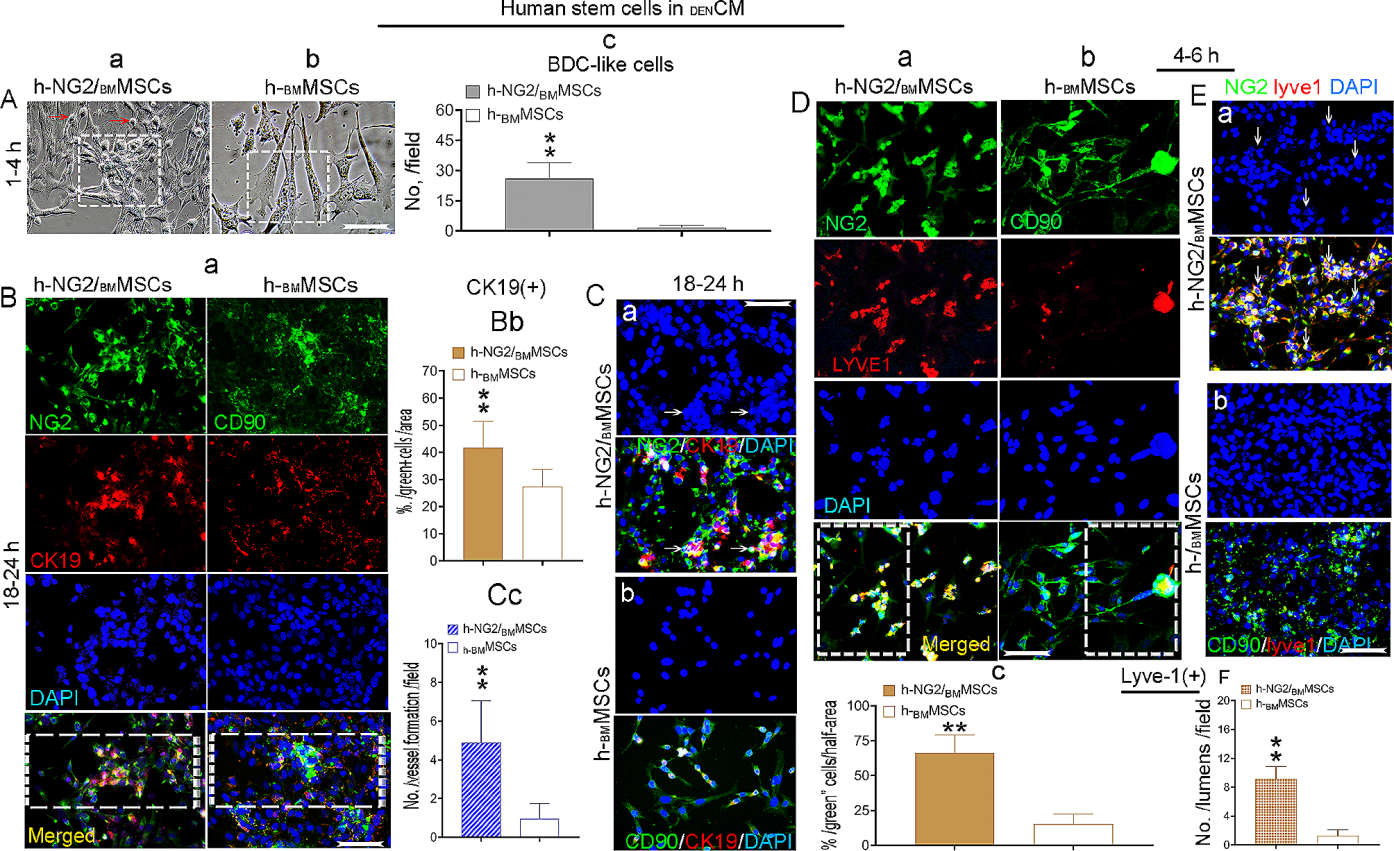
Potential of h-NG2/_BM_MSCs to develop BDCs and their unique ability to directly differentiate into sinusoidal cells and structures in response to DEN-induced liver injury cues. (Aa-c) During 1–4 h of culture in _DEN_CM, BDC-like morphological changes were observed in culturing h-NG2/_BM_MSC cells that differentiated from h-NG2/_BM_MSCs **(a**, red arrows, box), but few similar changes were observed in cells that differentiated from parental h-_BM_MSCs **(b**, box), and the number of changed cells per image field was analyzed **(c**, *n* = 10). **(Ba, b)** Double IF staining of CK19^+^ cells **(a**, red, second panels) covered with h-NG2/_BM_MSCs (NG2, green, top panel) or h-_BM_MSCs (CD90, green, top panel) and analysis of the percentage of developed CK19^+^ cells/image half-field from merged cells **(b**, brown, boxes) during 18–24 h of culture in _DEN_CM, *n* = 6. **(Ca-c)** Double IF staining revealed that during the 18–24 h period in _DEN_CM cultures, the CK19^+^ cells developmed from h-NG2/_BM_MSCs formed obvious vessel-like structures (**a**, merged, arrows) that were not observed in the parental h-_BM_MSCs **(b)**, and quantification was performed **(c**, *n* = 6). **(Da-c)** Double IF staining of h-NG2/_BM_MSCs (NG2, green, **a**) and h-_BM_MSCs (CD90, green, **b**) covered with Lyve-1^+^ cells (red) after 4–6 h culture periods in _DEN_CM and quantification of the merged cells in Da, b **(c**, boxes), *n* = 6. **(Ea, b)** In the _DEN_CM cultures, Lyve-1^+^ cell (red)-generated from h-NG2/_BM_MSC cells (green) formed vessel-like structures (**a**, arrows) and this phenomena was not appeared in the h-_BM_MSCs **(b)**. **(F)** Quantification of the data in E (*n* = 6). Scale bar = 200 μm for all images. At least three independent experiments were performed, and the data are presented as the means ± SDs; **p < 0.001 compared with h-_BM_MSCs

In order to assess whether soluble signals from damaged livers could promote functional differentiation, cells were plated at a low density (1 × 10^5^) in the presence of _DEN_CM, after which the numbers of cholangiocyte and LSEC lineage cells that developed from the two types of cells were compared. After 1–4 h, the BDC-like morphological cells (arrows) devedoped from the h-NG2/_BM_MSCs (Fig. 7Aa) increased dramatically (Fig. 7Aa, a box), while the morphology of the parental h-_BM_MSCs exhibited little change at this time point (Fig. 7Ab, c, boxes). Double IF staining revealed that the h-NG2/_BM_MSC-sourced BDC-shaped cells (NG2, green) were significantly more strongly stained with CK19 (red, second panels) than the parental h-_BM_MSCs (CD90, green). Approximately 41.71%±9.73% of the CK19^+^ cells developed from h-NG2/_BM_MSCs in _DEN_CM, which was significantly greater than the percentage that developed from parental h-_BM_MSCs (27.34%±6.37%; Fig. 7Ba, merged, boxes, b) in the same _DEN_CM. These data suggest that h-NG2/_BM_MSCs quickly promoted liver BDC repair. Interestingly, in those cultures, this staining also catched the CK19^+^ cells (red) formed significant number of vessel-like structures that developed from the h-NG2/_BM_MSCs (green, arrows, Fig. 7Ca), which was not observed in the h-_BM_MSCs (Fig. 7Cb, c), suggesting that h-NG2/_BM_MSCs had the capacity to promote the reconstruction of bile duct structures in diseased livers.

We next determined whether sinusoidal cells were more likely to develop from h-NG2/_BM_MSCs in response to DEN-induced cues by performing double IF staining again. A significantly greater number of Lyve-1^+^ cells (red) that developed from h-NG2/_BM_MSCs (NG2, green; Fig. 7Da, 66.38%±12.8%) than from parental h-_BM_MSCs (CD90, green, 15.21% ±7.25%; Fig. 7Db, **c**, merged; boxes) was detected after 4–6 h of culture in _DEN_CM. More interestingly, in these cultures, we detected that the soluble signals from _DEN_CM promoted the formation of a sinusoidal lumen-like structure in Lyve-1^+^ cells that developed from h-NG2/_BM_MSCs (Fig. 7Ea, arrows), but few of these structures formed from parental h-_BM_MSCs (Fig. 7Eb), representing a dramatic difference (Fig. 7F) and suggesting that h-NG2/_BM_MSCs also had the ability to contribute to mature cholangiocyte generation and self-renewing LSEC structures because of their unique biological properties. These new findings indicate that fast improvement of host BDC and LSEC cell repair by h-NG2/_BM_MSC transplantation may occur through direct functional differentiation of these cells into BDCs, particularly sinusoidal cells, which may initiate regeneration and functional recovery.

## Discussion

Cellular therapies are becoming increasingly important in developing treatments for liver disorders, and mesenchymal dermal tissue-sourced stem cells (MSCs), such as MSCs generated from bone marrow (_BM_MSCs) [41], adipose tissue [42], the umbilical cord [43], the liver [23, 44], and multiple organ tissues [45], are the most common adult stem cells used for this purpose. However, the heterogeneity of these MSCs, which decreases their efficacy, limits their use in regenerative medicine. Therefore, the isolation of distinct effective cell populations will lead to effective purity that enhances the efficacy for treating diseases including liver insults. We built upon this initial work using a method to isolate an NG2^+^ cell subset from heterogeneous _BM_MSCs (NG2/_BM_MSCs) cultures to show the value of these new progenitors for enhancing therapeutic efficacy. This study provides novel insights into the complex heterogeneity of _BM_MSCs in terms of their biological and functional characteristics in an animal model of liver fibrosis/cirrhosis induced by DEN.

Using a modified Percoll gradient selection process known as PWPP [21, 23], NG2/_BM_MSCs were successfully isolated from animals, and importantly. This approach is also applicable for human marrow-derived MSCs (h-NG2/_BM_MSCs). The purity of NG2^+^ cells from both species reached 95-98%. In normal cultures, NG2/_BM_MSCs exhibit a larger flaky morphology with multiple processes and a proliferation potential similar to that of their in vivo counterparts [7], and this evidence is consistent with that of other cases [46, 47]. However, although the fluorescence intensity histograms of NG2/_BM_MSCs were similar to those of parental _BM_MSCs, we for the first time provided the notable differences between the two types of cells. For example, the rapidly dividing (Ki-67^+^) population of NG2-labeled _BM_MSCs (NG2/_BM_MSCs) was larger and more granular with higher SSEA-3 expression and likely generated smaller spindle-shaped cells which exhibited slowly dividing and lower expression of SSEA-3 than that of NG2/_BM_MSCs, suggesting more “stemness” potential than parential _BM_MSCs. This evidence is inconsistent with the literature [7] regarding immature or “stemness” issues, in contrast to what has been previously reported [7].

When infused cells into mice with the ongoing DEN-induced liver fibrotic/cirrhotic injury mouse model, NG2/_BM_MSCs had greater efficacy than parental _BM_MSCs in promoting tissue repair and functional recovery, which was associated with a reduction in the extent of *inflammation, fibrotic load*, increased numbers of endogenous cholangiocytes (CK19^+^), BDC-mediated hepatocyte regeneration and vessel-like reconstruction, *and regeneration of hepatocytes (Alb*^*+*^, *G6Pc*^*+*^*)* in lesioned areas of the damaged liver. The exposure of cells to medium conditioned by DEN-induced liver tissue (_DEN_CM) resulted in similar outcomes, suggesting that fate determination in NG2/_BM_MSCs can be sensitively modulated by pathological signals derived from injured livers. Importantly, also for the first time, this study reported that NG2/_BM_MSCs play a unique role in sinusoidal or SLEC lineage cell differentiation in both DEN-induced diseased liver niche and in the presence of _DEN_CM cues that were not observed for parental _BM_MSCs, suggesting that NG2/_BM_MSCs may serve as novel off-liver progenitors of LSECs for liver regeneration initiation and may also be novel specific seed cells for liver tissue engineering.

The in vivo correlation of isolated NG2/_BM_MSCs is currently unclear. This study showed that NG2/_BM_MSCs share several characteristics with pericytes (PCs), which are known to possess stem cell properties. For example, these cells express both NG2 and PDGFR-β (Suppl. Figs. S1C; S3E) [48], which is consistent with the characteristics reported in the literature [7]. However, whether NG2^+^ cells can generate multiple cell types is currently unclear. In the present study, we showed that NG2/_BM_MSCs not only were highly motile (Fig. 4A, B) and proliferative both in injured liver lesions (Fig. 6B) and in the diseased cues (Fig. 2D-F) but were also able to differentiate into multiple functional cell lineages, such as BDCs, ECs, and LSECs, to reconstruct the biliary tree, blood and sinusoidal vessels, leading to endogenous hepatocyte regeneration. These findings would be an mechanic explanation that NG2/_BM_MSCs have greater potential to exert these effects than parental _BM_MSCs. *Furthermore, the superior effects of NG2/*_*BM*_*MSCs to parental*_*BM*_*MSCs in reducing inflammatory infiltration and fibrosis *(Suppl. Fig. S2A, B) and balancing immune response (not shown) may also be closely related to the mechanism of promoting functional recovery in the intact adult liver [23]. Our previous findings also support the present hypothesis that NG2^+^ cells directly affect repair. For instance, transplantation of liver-derived NG2^+^ cells increased endogenous hepatocyte regeneration in lesion areas of liver fibrosis/cirrhosis [8, 49]. Transplantation of NG2^+^ cells sourced from the spinal cord resulted in enhanced axonal survival in regions of spinal cord injury [50].

The unique property of NG2/_BM_MSCs in LSEC lineage differentiation for supporting regeneration should be a relatively important discovery in this study. One hypothesis for interpreting the mechanism is that direct LSEC differentiation from NG2/_BM_MSCs to reconstruct sinusoidal structures and initiate overall regeneration [51] results in functional recovery, leading to therapeutic effects; this contribution is unique for NG2/_BM_MSCs because _BM_MSCs cannot complete this process. Furthermore, the core role of NG2^+^ cells (Fig. 4E, F) in maintaining the functions of _BM_MSCs in bile duct repair was also revealed in this study. Moreover, in the setting of degeneration, NG2^+^ cells fast homed to areas of insult and differentiate into functional cells, which may also contribute to diseased liver repair [52, 53]. Additionally, all these cellular products are characteristic of the hepatic epithelium, and our data may suggest that NG2/_BM_MSCs of mesodermal origin could also provide a beneficial environment.

The nature of the signals that mediate enhanced endogenous regeneration of NG2/_BM_MSCs also remains unknown but may include growth factors such as hepatocyte growth factor (HGF), Interlukin-6 (IL-6), and the CCAAT/enhancer binding proteins (C/EBPs) [8, 54, 55], all of which are implicated in modulating liver tissue repair. Whether NG2^+^ cells promote functional differentiation from endogenous hepatic progenitor cells (HPCs) or promote the survival of existing functional hepatic cells and how long the donor cells would survive in the diseased liver niche requires additional investigation. Their identification will reveal important targets for future therapeutic approaches to stimulate cirrhotic liver repair and regeneration.

## Conclusions

In summary, this present study defines four issues. First, a PPWP can be used for the isolation of NG2^+^ cell subset from heterogeneous marrow MSC cultures. Clinically, this PPWP strategy should have a significant impact on the manufacturing of MSC therapies because MSCs must be expanded ex vivo for most clinical applications due to their rarity in tissues. Thus, this isolation strategy could aid in the selection of sygeneic/autologous or allogeneic donor MSCs harvested from patients or from cell banks to evaluate the quality. Moreover, as this approach can be also used to generate NG2^+^ cells from multiple adult organ-sourced MSC cultures (not shown), all these could be new tools for enhancing the therapeutic efficacy in clinical trials not only beneficial for _BM_MSC therapy on recipients with liver fibrosis/cirrhosis but may also for those with other diseases. Second, ex vivo-expanded NG2/_BM_MSCs have different biological and functional features compared to parental _BM_MSCs. In normal cultures, larger flaky NG2/_BM_MSCs showed more “stemness” than smaller spindle-shaped _BM_MSCs; in injured livers or in response to injured liver cues, NG2/_BM_MSCs are more powerful than parental _BM_MSCs in inducing the differentiation of BDCs and ECs, and promoting BDC-mediated hepatocyte regeneration to support injured liver regeneration; *additionally, NG2/*_*BM*_*MSCs also have advantages over parental*_*BM*_*MSCs in improving pathological condition* to support functional recovery. Therefore, this more pure NG2/_BM_MSCs could be a novel cell subset and important advance for _BM_MSC or MSC cell-based therapy to patients with liver or other disorders. Third, NG2/_BM_MSCs play a core role in supporting the ability of _BM_MSCs to repair injured bile ducts, indicating that the activity of NG2^+^ cells within _BM_MSCs may be critical for the functions of _BM_MSCs. Fourth, for the first time, the unique capacity of NG2/_BM_MSCs in LSEC differentiation to reconstruct sinusoidal structures in the injured liver was revealed, raising two important issues: (1) NG2/_BM_MSCs are hypothesized to function as novel off-liver progenitors of LSECs to initiate injured liver regeneration, and NG2/_BM_MSCs are not dependent on _BM_MSCs. (2) The progenitors are considered novel specialized seed cells for liver tissue engineering. However, further valuable insights into issues that the cellular and molecular pathways that mediate recovery from liver insults, *whether treatments with syngeneic/allogeneic or autologous or xenogeneic NG2/*_*BM*_*MSCs in this animal model exhibit distinctive individual characteristics; and how immune responses occur in these situations need to be further compared. However, some limitations in this study should also be taken account. For example, NG2/*_*BM*_*MSC subset sourced human and animals sounds existing bias in the DEN mouse model and probably more relative animal models are required to do the evaluation*. Overall, these data are fundamentally important as new advances and novel therapeutic tools in future clinic, *in particular, to the utilization of *_*BM*_*MSCs or may other biological system-sourced MSCs, this PPWP strategy would have potentially numerous applications*.

### Electronic supplementary material

Below is the link to the electronic supplementary material.

**Supplementary Material 1**: **Supplemental fig. S1** use of the PPWP for isolating NG2^+^ cells from cultures of _BM_MSCs and assessment of several biological features. (A-Ba-d) Marrow aspiration (a) followed by plating into dishes (b) for the primary culture of _BM_MSCs (Step 1). After 7–10 days of passage two cultures (P2), the cells were plated on a Percoll gradient to obtain a fraction (c) and then cultured again for approximately 3 days; this process was repeated 1–2 times [repear-step (1)-(3)], depending on the cell quality (Step 2). After 1 week of culture again with the first two fractions, the assumed NG2^+^ cells were isolated and ready to use after passages (d, Step 3), and pink cell-like cartoons (Bd) indicate assumed NG2^+^ cells. (C) Surface markers of m-_BM_MSCs were analyzed using FCM. (D) The differentiation of osteogenic and adipogenic cells was monitored by the formation of lipid droplets and osteocalcin. *n* = 3/type experiment. Scale bars = 200 μm for the images in B and 100 μm for the images in D.

**Supplementary Material 2**: **Supplemental fig. S2** pathological changes in the DEN model after cell treatment and the EC cell differentiation potential of m-NG2/_BM_MSCs in response to injured liver cues (_DEN_CM). *(Ai-ii, iv)*. *H&E staining for inflammatory infiltration in subgroup liver sections*(i)*and quantificative scores *(ii, iv). (B) *Massion trichrome *(*MT*) *staining for fibrotic collagen *(*blue*, i) *and quantification scores for fibrosis in subgroup liver sections *(ii, iv). *The score evaluation was based on NAS scores (Mat/Met)*. (Ca-c) Double IF staining of NG2^+^ (green) or CD9^+^ (red) cells stained with CD31 (red/green) for m-NG2/_BM_MSCs (a) and m-_BM_MSCs (b); arrows show that CD31^+^ cells formed vessel-like structures (Ca) that were not detected in m-_BM_MSCs, and quantification (c) of the number of merged cells from C (merged, per quarter area/boxes). (Da-c) The same analysis as CD31 was used for vWf^+^ cell staining in both m-NG2/_BM_MSCs and m-_BM_MSC cells (a, b), and the data were quantified (c, *n* = 6). (E-F) Analysis of the number of vWf^+^ cells that developed from m-NG2/_BM_MSCs formed vessel-like structures (Ca, arrows, E); this phenomenon was also observed in m-_BM_MSCs (Cb, F). At least three independent experiments were performed, and the data are presented as the means ± SDs. Scale bar = 200 μm. #*p < 0.05 compared with either DEN or m-_BM_MSCs

**Supplementary Material 3**: **Supplemental fig. S3**. characterization of ex vivo-expanded h-NG2/_BM_MSCs using FCM and IF staining. (A) FCM was used to analyze the percentage of NG2^+^ cells in heterogeneous cultures of h-_BM_MSCs (the arrow indicates the approximate percentage of NG2^+^ cells within the h-_BM_MSC cultures). (Ba, b) Using the PPWP, a fraction was obtained (a, an arrow indicates the NG2^+^ cell proportion), and expanded cultures were generated from the fraction (b). (Ca, b) IF staining (red, a) and FCM (b) were used to label passage 2 cultures of ex-vivo-expanded of h-NG2/_BM_MSCs, and both methods showed greater purity (> 95%), *n* = 3/technique; scale bar = 200 μm. (Da, b) *IF staining for endogenous G6Pc expression in DEN liver 4 weeks after cell treatment*(a, b), *and quantificative analysis for numbers*(c)*and x-fole*(d)*changes, n = 6; scale bar = 200 μm; At least three independent experiments were performed, and the data are presented as the means ± SDs*. **p *< 0.001 compared with h-*_*BM*_*MSCs.*(E) FCM also showed that h-NG2/_BM_MSCs share some markers with parental h-_BM_MSCs; *n* = 3

## Data Availability

Data are contained within the article.

## Abbreviations

MSCs: mesenchymal stem cells
NG2: Neuroglial antigen 2
h-NG2/_BM_MSCs: Human donor _BM_MSC-sourced NG2^+^ cells
DEN: Diethylnitrosamine
ECM: Extracellular matrix
PCs: Pericytes
CNS: The central nervous system
m_− BM_MSCs: Adult mouse-derived _BM_MSCs
m-NG2/_BM_MSCs: NG2^+^ cells from adult mouse _BM_MSC cultures
h_− BM_MSCs: Human bone marrow-derived MSCs
h-NG2/_BM_MSCs: NG2^+^ cells from human _BM_MSC cultures
PPWP: Percoll–Plate–Wait procedure
CFSE: Carboxyfluorescein diacetate succinimidyl ester
FCS: Fetal Calf Serum
SIP: Stock isotonic Percoll
PLL: Poly-L-lysine
SSEA-3: Stage-specific embryonic antigen-3
vWf: Von Willebrand factor
LYVE-1: Lymphatic vessel endothelial hyaluronan receptor-1
LSECs: Liver sinusoidal endothelial cells
ɑ-SMA: Alpha-smooth muscle actin
HSCs: Hepatic stellate cells
FCM: Flow cytometry
PDGFR-β: Platelet-derived growth factor receptor-beta
IHC: Immunohistochemistry
IF: Immunofluorescence
H&E: Hematoxylin and eosin
MT: Masson’s trichrome
NGS: Normal goat serum
BDCs: Bile duct cells
ECs: Endothelial cells
TBIL: Total bilirubin
DBiL: Direct bilirubin
IBiL: Indirect bilirubin
ALP: Alkaline phosphatase
ALT: Alanine transaminase
AST: Aspartate transaminase
LDL: Low-density lipoprotein
Alb: Albumin
G6Pc: Glucose-6-phosphatase
_DEN_CM: CM generated from liver tissues at 6–7 weeks post-DEN liver
Ctrl-CM: Conditioned medium (CM) from normal cultures
Post-DEN: After DEN administration
MACS: Magnetic activated cell sorting
