# Supplementary figures and images for: New advances of NG2-expressing cell subset in marrow mesenchymal stem cells as novel therapeutic tools for liver fibrosis/cirrhosis
Source: Stem Cell Res Ther. 2024 Jul 6;15:199. doi: 10.1186/s13287-024-03817-x (PMC11227708; doi:10.1186/s13287-024-03817-x)

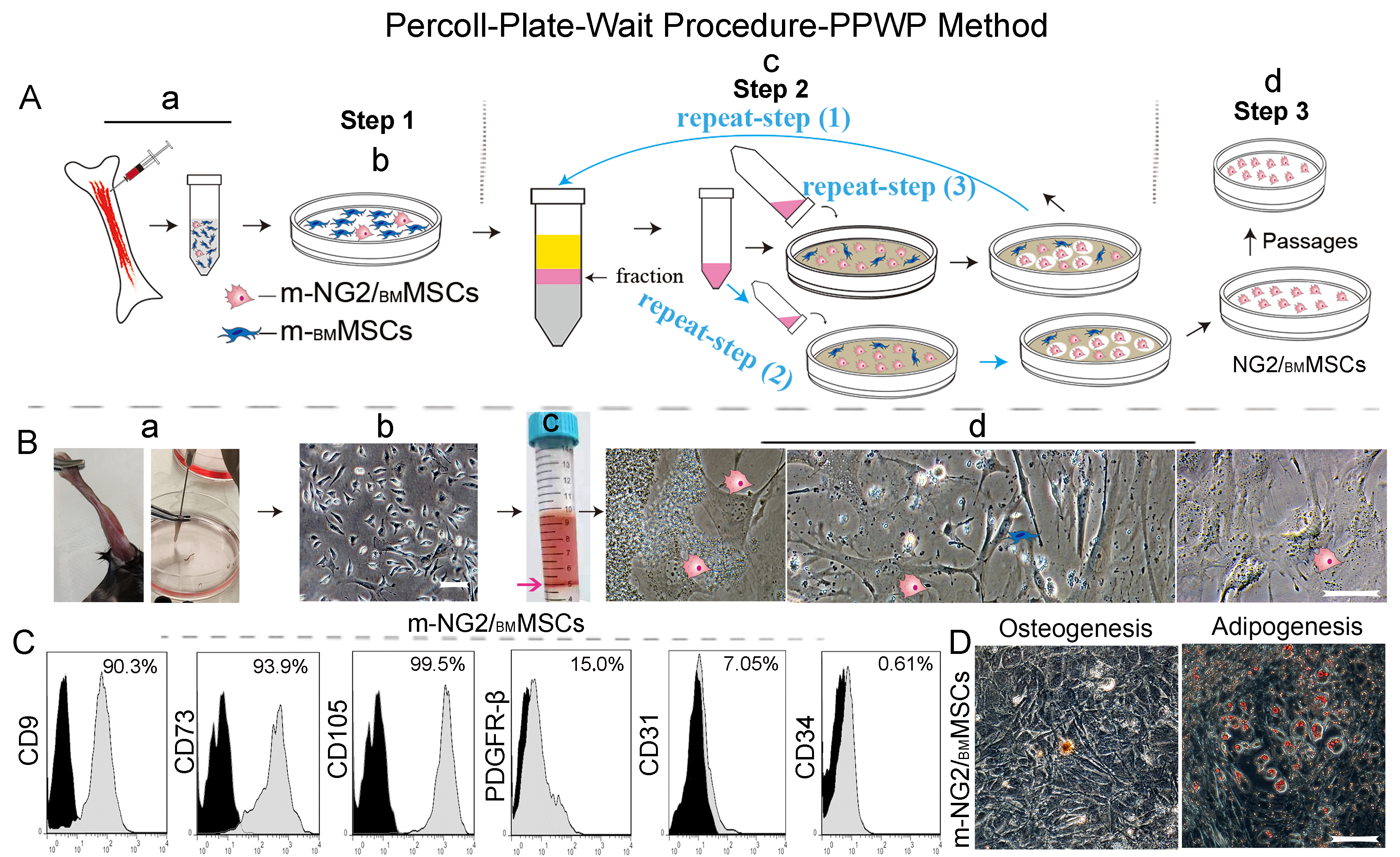

Supplement: Supplementary file 1 — Supplementary Material 1: Supplemental fig. S1 use of the PPWP for isolating NG2+ cells from cultures of BMMSCs and assessment of several biological features. (A-Ba-d) Marrow aspiration (a) followed by plating into dishes (b) for the primary culture of BMMSCs (Step 1). After 7–10 days of passage two cultures (P2), the cells were plated on a Percoll gradient to obtain a fraction (c) and then cultured again for approximately 3 days; this process was repeated 1–2 times [repear-step (1)-(3)], depending on the cell quality (Step 2). After 1 week of culture again with the first two fractions, the assumed NG2+ cells were isolated and ready to use after passages (d, Step 3), and pink cell-like cartoons (Bd) indicate assumed NG2+ cells. (C) Surface markers of m-BMMSCs were analyzed using FCM. (D) The differentiation of osteogenic and adipogenic cells was monitored by the formation of lipid droplets and osteocalcin. n = 3/type experiment. Scale bars = 200 μm for the images in B and 100 μm for the images in D. [file 13287_2024_3817_MOESM1_ESM.tif]

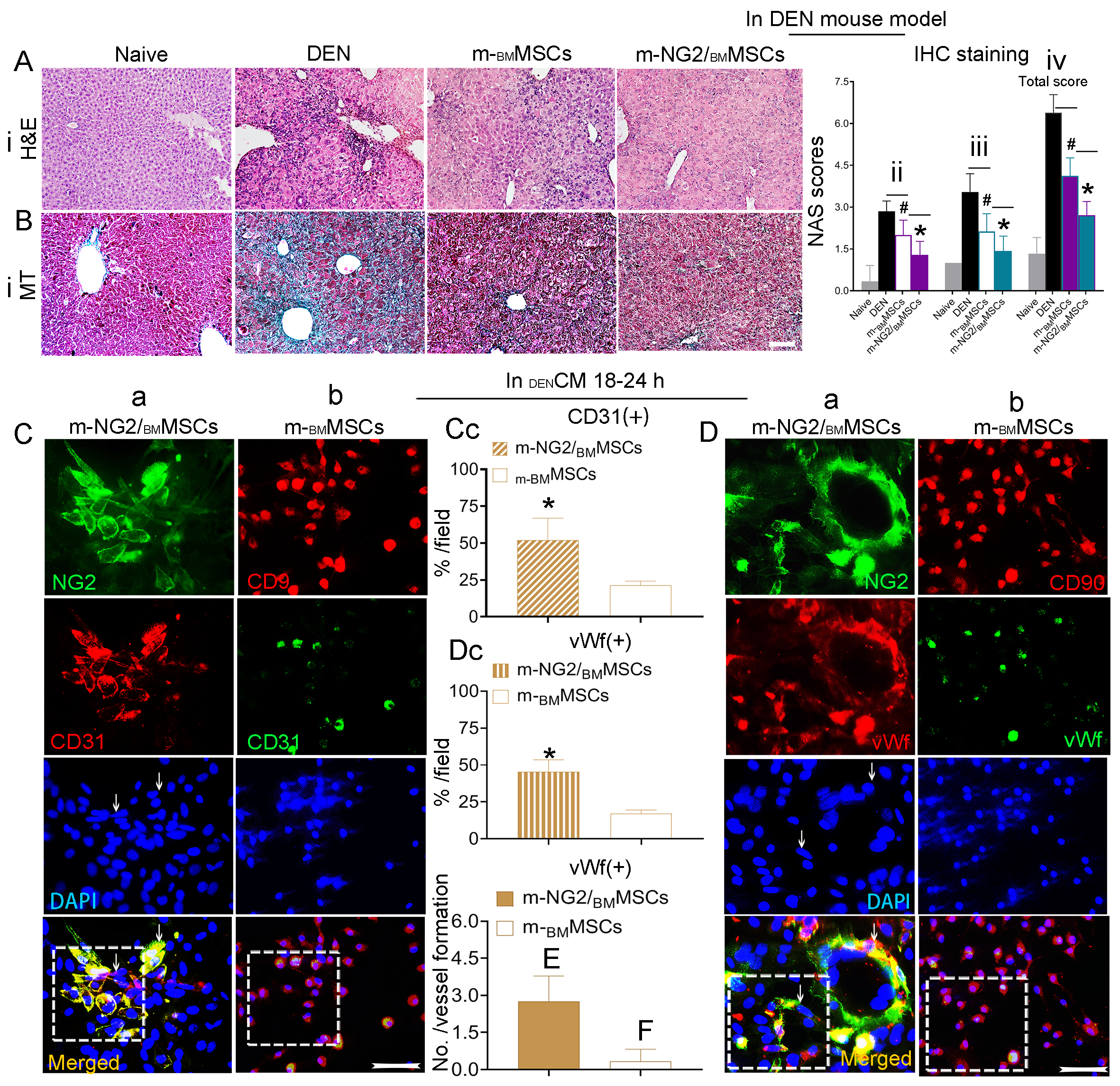

Supplement: Supplementary file 2 — Supplementary Material 2: Supplemental fig. S2 pathological changes in the DEN model after cell treatment and the EC cell differentiation potential of m-NG2/BMMSCs in response to injured liver cues (DENCM). (Ai-ii, iv). H&E staining for inflammatory infiltration in subgroup liver sections(i)and quantificative scores (ii, iv). (B) Massion trichrome (MT) staining for fibrotic collagen (blue, i) and quantification scores for fibrosis in subgroup liver sections (ii, iv). The score evaluation was based on NAS scores (Mat/Met). (Ca-c) Double IF staining of NG2+ (green) or CD9+ (red) cells stained with CD31 (red/green) for m-NG2/BMMSCs (a) and m-BMMSCs (b); arrows show that CD31+ cells formed vessel-like structures (Ca) that were not detected in m-BMMSCs, and quantification (c) of the number of merged cells from C (merged, per quarter area/boxes). (Da-c) The same analysis as CD31 was used for vWf+ cell staining in both m-NG2/BMMSCs and m-BMMSC cells (a, b), and the data were quantified (c, n = 6). (E-F) Analysis of the number of vWf+ cells that developed from m-NG2/BMMSCs formed vessel-like structures (Ca, arrows, E); this phenomenon was also observed in m-BMMSCs (Cb, F). At least three independent experiments were performed, and the data are presented as the means ± SDs. Scale bar = 200 μm. #*p < 0.05 compared with either DEN or m-BMMSCs [file 13287_2024_3817_MOESM2_ESM.tif]

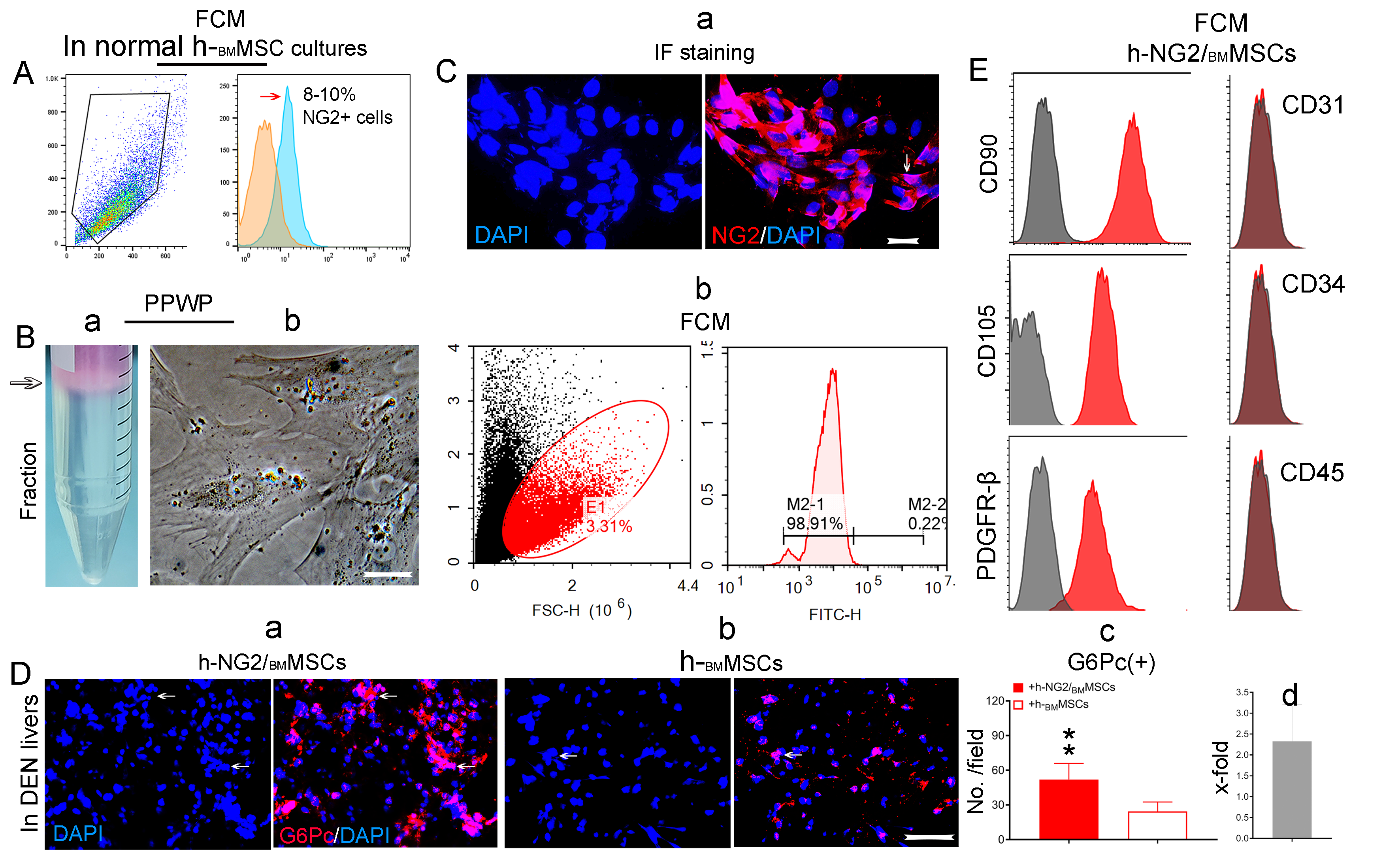

Supplement: Supplementary file 3 — Supplementary Material 3: Supplemental fig. S3. characterization of ex vivo-expanded h-NG2/BMMSCs using FCM and IF staining. (A) FCM was used to analyze the percentage of NG2+ cells in heterogeneous cultures of h-BMMSCs (the arrow indicates the approximate percentage of NG2+ cells within the h-BMMSC cultures). (Ba, b) Using the PPWP, a fraction was obtained (a, an arrow indicates the NG2+ cell proportion), and expanded cultures were generated from the fraction (b). (Ca, b) IF staining (red, a) and FCM (b) were used to label passage 2 cultures of ex-vivo-expanded of h-NG2/BMMSCs, and both methods showed greater purity (> 95%), n = 3/technique; scale bar = 200 μm. (Da, b) IF staining for endogenous G6Pc expression in DEN liver 4 weeks after cell treatment(a, b), and quantificative analysis for numbers(c)and x-fole(d)changes, n = 6; scale bar = 200 μm; At least three independent experiments were performed, and the data are presented as the means ± SDs. **p < 0.001 compared with h-BMMSCs.(E) FCM also showed that h-NG2/BMMSCs share some markers with parental h-BMMSCs; n = 3 [file 13287_2024_3817_MOESM3_ESM.tif]
